# The NSL complex is required for piRNA production from telomeric clusters

**DOI:** 10.26508/lsa.202302194

**Published:** 2023-06-30

**Authors:** Shantanu S Iyer, Yidan Sun, Janine Seyfferth, Vinitha Manjunath, Maria Samata, Anastasios Alexiadis, Tanvi Kulkarni, Noel Gutierrez, Plamen Georgiev, Maria Shvedunova, Asifa Akhtar

**Affiliations:** 1 Max Planck Institute of Immunobiology and Epigenetics , Freiburg im Breisgau, Germany; 2 Spemann Graduate School of Biology and Medicine (SGBM), University of Freiburg, Freiburg im Breisgau, Germany; 3 Faculty of Biology, University of Freiburg, Freiburg im Breisgau, Germany

## Abstract

The NSL complex is a transcriptional activator. Germline-specific knockdown of NSL complex subunits NSL1, NSL2, and NSL3 results in reduced piRNA production from a subset of bidirectional piRNA clusters, accompanied by widespread transposon derepression. The piRNAs most transcriptionally affected by NSL2 and NSL1 RNAi map to telomeric piRNA clusters. At the chromatin level, these piRNA clusters also show decreased levels of H3K9me3, HP1a, and Rhino after NSL2 depletion. Using NSL2 ChIP-seq in ovaries, we found that this protein specifically binds promoters of telomeric transposons *HeT-A*, *TAHRE*, and *TART*. Germline-specific depletion of NSL2 also led to a reduction in nuclear Piwi in nurse cells. Our findings thereby support a role for the NSL complex in promoting the transcription of piRNA precursors from telomeric piRNA clusters and in regulating Piwi levels in the Drosophila female germline.

## Abstract (precis)

Germline knockdown of the NSL complex leads to transposon derepression, particularly of telomeric transposons. NSL2 binds promoters of telomeric transposons and its depletion leads to alterations in the chromatin state of multiple telomeric piRNA clusters.

## Introduction

Transposable elements (TEs) are DNA sequences which are capable of changing their position within the genome using either “copy and paste” or “cut and paste” mechanisms. TEs comprise 22% of the *Drosophila melanogaster* genome (Kapitonov & Jurka, 2003) and are located predominantly in or adjacent to heterochromatic regions such as pericentromeres (Yamanaka et al, 2014). The Drosophila genome also houses special classes of TE occurring at the ends of chromosomes. Telomeres are structures which cap linear chromosome ends and protect them from attrition because of progressive shortening during DNA replication cycles (O’Sullivan & Karlseder, 2010). Furthermore, telomeres prevent chromosome ends from being recognized by the double-stranded break repair machinery, which could otherwise generate end-to-end chromosome fusions driving genomic instability (Doksani & de Lange, 2014). In humans, telomeres are maintained through the activity of a specialized DNA repeat-adding enzyme termed telomerase. However, telomerase is absent in the *Diptera* lineage, necessitating the emergence of a distinct telomere maintenance mechanism (Radion et al, 2018). The telomeres of Drosophila consist of three classes of transposons (*HeT-A*, *TART*, and *TAHRE*, collectively referred to as HTTs) arranged in unidirectional arrays. The HTTs’ unique ability to replicate and insert at the ends of chromosomes has been co-opted for telomere maintenance in Drosophila (Pardue et al, 2005). Therefore, whereas many TE classes are considered either functionally inert or are transiently mobilized to shuffle the genome for accelerating evolution, telomeric TEs are paradoxically required to maintain genome integrity (Mérel et al, 2020).

TEs can pose a threat to the integrity of the host genome. As a result, organisms have evolved strategies to suppress transcription of TEs. The PIWI-interacting RNA (piRNA) pathway is the primary mechanism used by metazoans to transcriptionally repress active TEs (for two recent reviews, see Onishi et al [2021] and Parhad & Theurkauf [2019]). piRNA clusters are genomic regions which serve as templates for RNA transcripts known as piRNA precursors that are processed into short sequences known as piRNA. Chromosome locations with a high density of TEs and TE fragments have been designated as piRNA clusters and are considered a major source of piRNA (Brennecke et al, 2007). Two main types of transposon clusters exist in fly ovaries: the germline dual-strand clusters and the somatic (follicle cell-specific) uni-strand clusters (Li et al, 2009; Malone et al, 2009). Another way to classify transposon clusters is into Group 1 (germline), Group 2 (mixed), and Group 3 (somatic) (Li et al, 2009).

Dual-strand piRNA cluster transcription in Drosophila is complicated by the fact that it is driven by a combination of canonical (promoter-dependent) and noncanonical (promoter-independent) transcription. All major germline piRNA clusters rely on noncanonical transcription driven by assembly of the Rhino, Deadlock and Cutoff complex, which does not appear to be conserved outside of the Drosophilidae (Klattenhoff et al, 2009; Pane et al, 2011; Mohn et al, 2014; Gamez et al, 2020). Rhino is an HP1 paralog which can recognize H3K9me3 using its chromodomain and is only expressed in the germline (nurse cells and oocytes) but not in somatic follicle cells of the ovary (Mohn et al, 2014; Sumiyoshi et al, 2016). The Rhino, Deadlock and Cutoff complex recruits a unique transcription machinery composed of Moonshiner, TFIIA-S, and TRF2 to dual-strand piRNA clusters. Moonshiner is a paralogue of RNA polymerase II-associated basal transcription factor IIA-L (TFIIA-L), which permits transcription initiation throughout the body of the piRNA cluster (Andersen et al, 2017). In addition, several major piRNA clusters including *cluster 42AB* and *cluster 38C1* are flanked by canonical promoters exhibiting prominent Pol II and TBP ChIP-seq peaks (Parhad et al, 2020). Although Rhino is predominantly found along the body of piRNA clusters, Rhino and Cutoff binding are also observed at these flanking promoters (Parhad et al, 2020). Although Rhino is not required for canonical transcription, it likely participates in it at steady state (Andersen et al, 2017). The protein Maelstrom has been found to be important for repressing any canonical transcription enabled by Rhino (Chang et al, 2019). Interestingly, artificially tethering Maelstrom using λN-box B repressed the transcription of a CG14072-luc reporter in cultured ovarian somatic cells (Onishi et al, 2020). However, Maelstrom loss in the ovary has little effect on H3K9me3 accumulation at most transposons except *HeT-A*, *TAHRE,* and *TART* (Sienski et al, 2012; Chang et al, 2019). *mael*^*M391/r20*^ null mutants exhibit profound up-regulation of RNA coding for telomeric transposons *HeT-A* (∼360-fold) and *TAHRE* (∼49-fold) and >twofold decrease in H3K9me3 ChIP signal at seven out of eight telomeric piRNA clusters (Chang et al, 2019). *mael*^*M391/r20*^ ovaries also showed increased H3K4me3 signal at derepressed transposons, suggesting that Maelstrom influences the balance between canonical and noncanonical transcription (Chang et al, 2019). Levels of canonical and noncanonical piRNA transcription appear to also be balanced through the master regulator Cutoff (Parhad et al, 2020). However, the full extent of the interplay between the different modes of piRNA cluster transcription is currently unknown, in particular, at telomeric piRNA clusters.

There are two major modes of piRNA-mediated silencing in Drosophila. Posttranscriptional silencing in the perinuclear nuage relies on PIWI family members Aub and Ago3. Aub and Ago3 are only expressed in the germline tissues, and not in the somatic follicle cells of the ovary (Li et al, 2009; Malone et al, 2009). Aub piRNA-induced silencing complexes (Aub–piRISCs) recognize cytoplasmic transposon mRNAs and cleave them using slicer activity. The cleavage products are converted to sense piRNAs which are loaded onto Ago3 (Czech et al, 2018). Ago3–piRISCs can in turn recognize and cleave transposon mRNAs, generating a combination of: (a) piRNAs which are subsequently loaded onto Aub in a loop termed the “ping-pong” cycle, and (b) an RNA product which participates in Zucchini (Zuc)-mediated piRNA biogenesis. In Drosophila, most of the piRNAs generated by Zuc are subsequently loaded onto Piwi (Czech et al, 2018). Association with piRNAs results in a conformational change which exposes a nuclear localization signal in Piwi, driving the import of loaded Piwi (Piwi–piRISCs) into the nucleus (Yashiro et al, 2018).

Transcriptional silencing, on the other hand, is mediated by Piwi, which is expressed both in germline and somatic ovary cells (Li et al, 2009; Malone et al, 2009; Klenov et al, 2011). Piwi–piRISCs identify nascent transposon transcripts by complementary base-pairing. Recent work identified a complex known as Pandas, SFiNX, PPNP or PICTS as an important cofactor in cotranscriptional silencing downstream of Piwi (Batki et al, 2019; Fabry et al, 2019; Murano et al, 2019; Zhao et al, 2019). Loss of Pandas/SFiNX/PPNP/PICTS complex components *panx* or *nxf2* leads to a significant reduction of H3K9me3 and derepression of Piwi-regulated transposons in both cultured ovarian somatic cells (Batki et al, 2019; Murano et al, 2019) and in ovaries (Fabry et al, 2019; Zhao et al, 2019). Association with dynein light chain Cut up/LC8 appears necessary for dimerization of the Pandas/SFiNX/PPNP/PICTS complex (Eastwood et al, 2021; Schnabl et al, 2021), which promotes single-stranded RNA binding in vitro and was hypothesized to enable the complex to tether nascent RNA to the underlying chromatin locus in vivo (Schnabl et al, 2021). However, the relationship between the Pandas/SFiNX/PPNP/PICTS complex and the dLsd1/Su(var)3–3 and Eggless/dSETDB1 enzymes responsible for modifying chromatin, and the precise sequence of events occurring during silencing are still largely unclear (Wang & Lin, 2021). Some data point to the involvement of the SUMO E3 ligase Su(var)2–10 in connecting Piwi and the Pandas/SFiNX/PPNP/PICTS complex with Eggless/dSETDB1 (Ninova et al, 2020). Intriguingly, analyses of ovaries of flies expressing Piwi protein lacking its nuclear localization signal (*piwi*^*Nt/*^*piwi*^*2*^) suggest that although transcriptional derepression of many transposon families takes place in *piwi*^*Nt/*^*piwi*^*2*^, only a fraction of TEs (including the HTTs) show a discernible concomitant change in H3K9me3 levels (Klenov et al, 2014).

piRNA-mediated silencing of telomeric transposons might therefore represent a special case. Indeed, telomeric piRNA clusters show several interesting features. The telomeric regions are self-silencing piRNA clusters, meaning their transcripts serve as both piRNA precursors and their own targets (Cacchione et al, 2020). The strong interdependencies between the three classes of telomeric TEs make it more helpful to think of them as a collective unit or assembly. *HeT-A* and *TART* elements contain promoters in their 3′ UTR responsible for driving the transcription of their neighboring element (Danilevskaya et al, 1997). *HeT-A* TEs are partial TEs which rely on the *TAHRE* and/or *TART* elements to supply an active reverse transcriptase for their transposition (Rashkova et al, 2002; Abad et al, 2004). On the other hand, the unique GAG-like protein encoded by *HeT-A* ORF1 might confer telomere specificity to the *TAHRE-* and *TART*-encoded reverse transcriptase (Fuller et al, 2010). Drosophila therefore possesses unique bifunctional telomeres which encode both the enzyme for their own maintenance and piRNAs which silence their own transcription (Savitsky et al, 2006). The competing demands to, on the one hand facilitate, and on the other hand repress, telomeric transcription therefore need to be carefully balanced to maintain genome integrity.

The transcription and maintenance of telomeric piRNA clusters relies on a unique chromatin state. In addition to Rhino, Drosophila telomeres are also characterized by the presence of HP1 variant HP1a (encoded by *Su(var)205*). The level of HP1a binding at the *HeT-A* promoter has been reported to be correlated to the level of *HeT-A* expression (Klenov et al, 2007). HP1a is required both for telomeric piRNA biogenesis and telomere maintenance (Teo et al, 2018). HP1a loss is associated with telomeric fusions in neuroblast cells, imaginal discs, and male meiotic cells (Fanti et al, 1998).

The nonspecific lethal (NSL) complex is a multi-subunit chromatin modifier consisting of NSL1, NSL2/dgt1, NSL3/Rcd1, MCRS2/Rcd5, MBD-R2, WDS, and MOF (Mendjan et al, 2006; Raja et al, 2010). It was shown to bind to the promoters and positively regulate the expression of more than 4,000 genes in the fly genome (Feller et al, 2012; Lam et al, 2012). The recruitment of the NSL complex to its target promoters results in the establishment of a nucleosome-free region. This is achieved by interaction with the NURF nucleosome-remodeling complex that ensures the strong positioning of the −1 and +1 nucleosomes (Lam et al, 2019). At its target genes, the NSL complex reduces transcriptional noise and maintains accurate TSS selection. The loss of the NSL complex results in reduced recruitment of the pre-initiation complex and RNA polymerase II (Pol II) to its targets (Lam et al, 2012). The gene-regulatory function of the NSL complex is conserved through to mammals, where it not only binds the promoters of constitutively active genes but also enhancers (Chelmicki et al, 2014). In humans, haploinsufficiency of the gene-encoding NSL1 (*KANSL1* in humans) results in a syndrome called Koolen–de Vries syndrome, characterized by developmental delay, intellectual disability, and other comorbidities (Koolen et al, 2012; Zollino et al, 2012).

Although the NSL complex has been relatively well characterized in the soma, its role in the germline remains unclear. Two screens, one performed in germline (Czech et al, 2013) and one in somatic ovarian cells (Muerdter et al, 2013), identified components of the NSL complex as affecting transposon up-regulation. In the present study, we describe the contribution of the NSL complex to the regulation of the piRNA pathway in the female germline. Depletion of the NSL complex in ovaries leads to derepression of transposons belonging to multiple families, with a strong effect on the *HeT-A*, *TAHRE*, and *TART* families. Knockdown of NSL2 leads to reduced production of piRNAs, particularly of those targeting HTTs, and a concomitant loss of H3K9me3 over several telomeric piRNA clusters. We also discover an unexpected binding of the NSL complex to the promoters present in the 3′-UTRs of the telomeric transposons. Furthermore, NSL2 RNAi showed epistasis with depletion of *mael*, with combined knockdown resulting in weaker *HeT-A* and *TAHRE* derepression than RNAi of each factor individually. In addition, we find that NSL2 depletion affects the levels of nuclear Piwi in nurse cells of the ovary. We suggest that the NSL complex influences piRNA production in two ways. First, by binding to the promoters of HTTs, the NSL complex may promote canonical transcription of telomeric piRNA precursors. Second, the NSL complex appears important to maintain adequate nuclear Piwi levels for maintenance of a repressive chromatin state at multiple piRNA clusters, including non-telomeric clusters.

## Results

### NSL complex depletion results in germline transposon derepression

**Figure 1.**
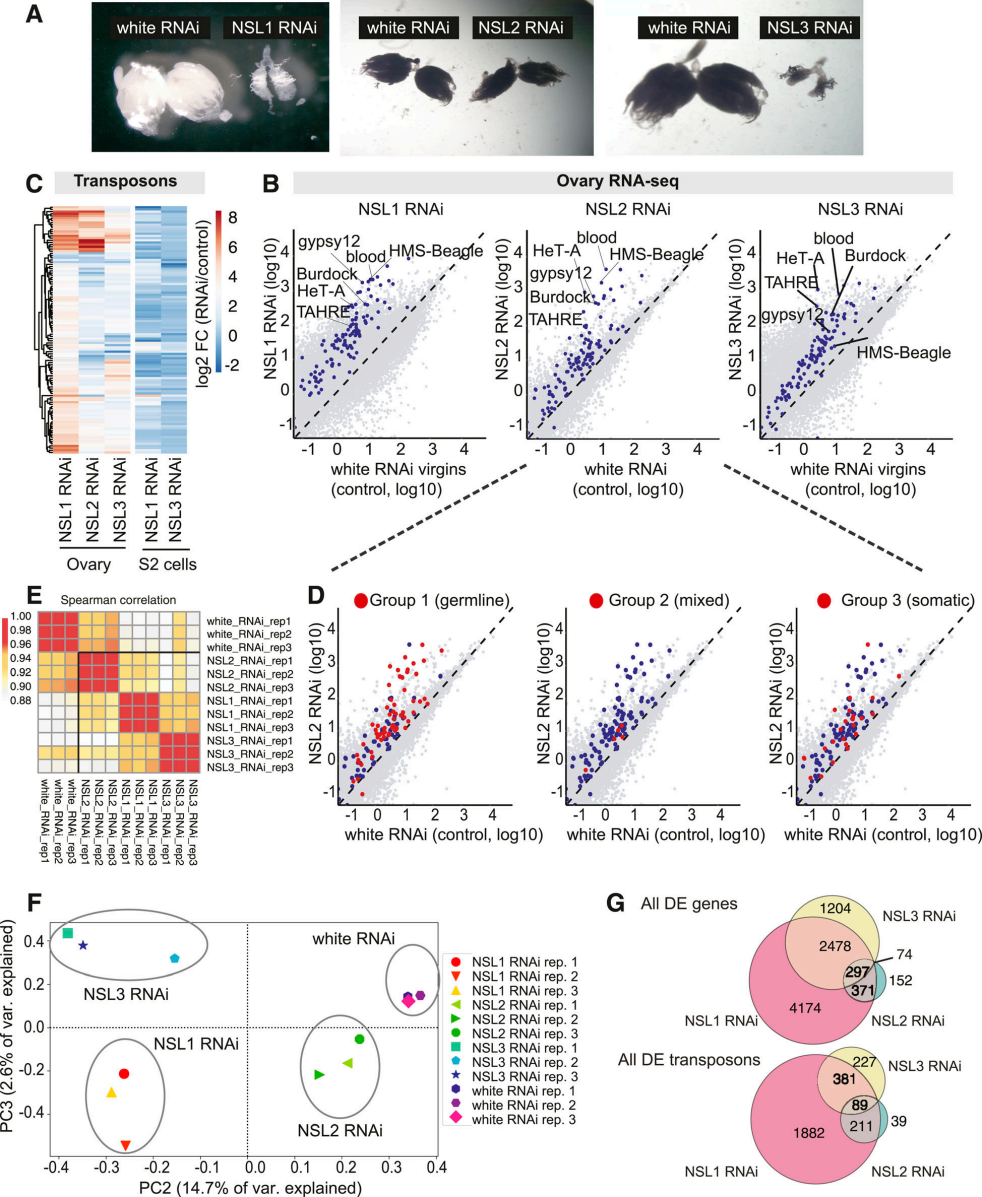
Germline depletion of NSL complex subunits results in transposon up-regulation. **(A)** Representative images of ovaries with germline (*nanos*-GAL4) knockdowns of *nsl1* (left), *nsl2* (middle), and *nsl3* (right). An ovary with knockdown of a control gene, *white*, is shown in each case. See also: Fig S1A. **(B)** Heatmap depicting log_2_ fold changes of RNA abundance of all transposon families upon NSL1, NSL2 or NSL3 RNAi in ovaries and NSL1 or NSL3 RNAi in S2 cells compared with control knockdowns. NSL1 and NSL3 RNAi in ovaries have been normalized to virgin white RNAi ovaries. NSL2 RNAi has been normalized to normal white RNAi ovaries. Data from S2 cells are derived from Gaub et al (2020). RNA-seq data represent the mean of three biological replicates, that is, ovaries collected from females from three separate crosses. **(C)** Scatterplots comparing steady-state RNA abundance between white RNAi and NSL1 RNAi (left), NSL2 RNAi (middle), and NSL3 RNAi (right) ovaries relative to controls. Virgin white RNAi ovaries are used as the control reference for NSL1 and NSL3 RNAi, and standard (nonvirgin) white RNAi ovaries are used as the control reference for NSL2 RNAi. Genes are coloured grey and transposons are coloured blue. Data represent the mean of three independent biological replicates. **(D)** Scatterplots comparing steady-state RNA abundance between white RNAi and NSL2 RNAi ovaries. Group 1 (left), Group 2 (middle), and Group 3 (right) transposons are highlighted in red. Transposons were classified according to Li et al (2009). Data represent the mean of three independent biological replicates. **(E)** Heatmap showing Spearman correlation of all RNA-seq datasets. **(F)** PCA plot of all RNA-seq datasets. **(G)** Overlap of all differentially expressed genes and transposon elements in RNA-seq upon NSL1, NSL2, and NSL3 knockdown in ovaries.

**Figure S1.**
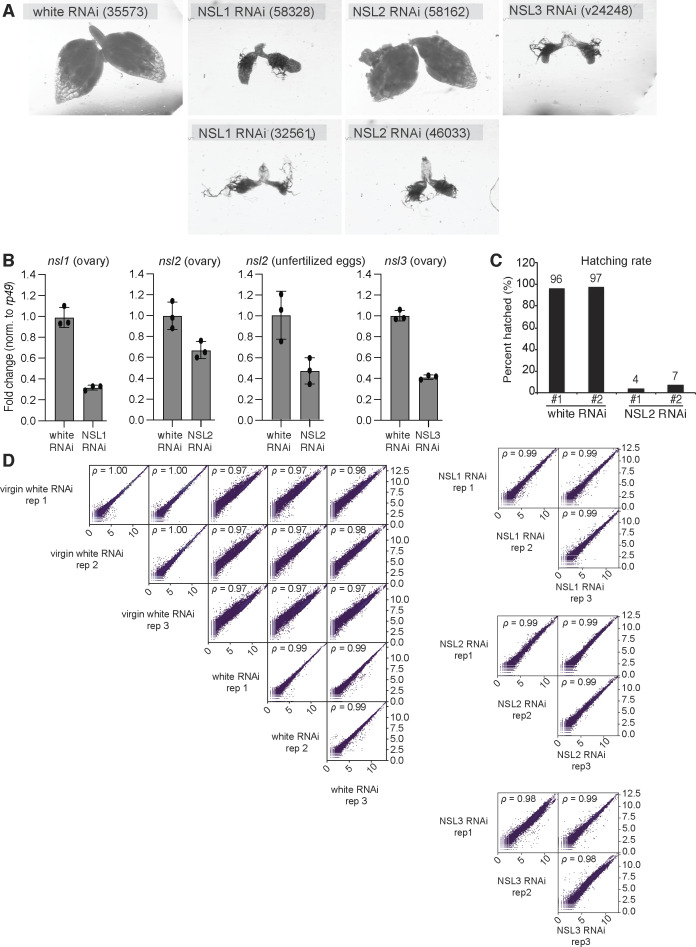
**(A)** Stereomicroscope photos of ovaries of flies subjected to *nanos*-GAL4 (25754)-driven RNAi-mediated depletion of white or NSL proteins. The Bloomington or VDRC stock center identifiers are indicated in brackets for each fly line. The same objective (magnification) was used for all images. **(B)** Barplots showing RT–qPCR fold changes for *nsl1* (left, ovary), *nsl2* (middle left, ovary), *nsl2* (middle right, unfertilized eggs), and *nsl3* (right, ovary) genes upon their respective germline knockdowns compared with controls. Data shown for *nsl1* and *nsl3* represent a comparison with white RNAi virgin ovaries as control to ensure a comparison between similar tissue morphology. Values were normalized to *rp49*. Values shown are an average of three biological replicates. Error bars represent SD. NSL1 RNAi (ovary) *P* = 8.67 × 10^−7^; NSL2 RNAi (ovary) *P* = 0.023; NSL2 RNAi (unfertilized eggs) *P* = 0.024; NSL3 RNAi (ovary) *P* = 1.05 × 10^−6^ (paired *t* test). **(C)** Barplot showing the percentage of hatched eggs laid by females with *nos*-GAL4-driven white RNAi and NSL2 RNAi for two independent crosses (#1 and #2). 439 and 495 eggs were counted for white RNAi. 339 and 259 eggs were counted for NSL2 RNAi. **(D)** Pairwise scatterplots showing correlations between RNA-seq replicates for each sample.

The NSL complex is an important transcriptional regulator in somatic tissues, but its function in the germline has not been explored in detail. Because full knockouts of individual NSL complex members are not viable, we used *nanos*-GAL4 to drive NSL-directed shRNAs specifically in the female germline. A line driving shRNA against the *white* gene in the germline using *nanos*-GAL4 was used as a control. Depletion of *nsl1* and *nsl3* resulted in atrophied ovaries, whereas NSL2 RNAi showed morphologically normal ovaries (Figs 1A and S1A). An RT-qPCR analysis from whole ovaries showed that NSL1 and NSL3 RNAi were more efficient, reducing the levels of *nsl1* and *nsl3* by 69% and 59%, respectively (Fig S1B). Meanwhile, levels of *nsl2* were reduced by 30–40% in NSL2 RNAi (Fig S1B). It is worth noting that these are likely underestimations of the knockdown because the tissue analyzed includes the somatic cells surrounding the germline cells, which do not express the shRNA. NSL2 RNAi showed a milder phenotype, which allowed the ovaries to develop and the flies to lay eggs. These eggs, however, hatch at severely reduced levels (5% of the control white RNAi; Fig S1C). Because the eggs laid by virgin flies would contain only the germline RNA contribution, we used them to quantify the efficiency of the knockdown. We found that *nsl2* levels were reduced by 54% in unfertilized eggs laid by NSL2 RNAi virgins (Fig S1B).

Because the NSL1 and NSL3 RNAi ovaries are rudimentary, we used ovaries isolated from white RNAi virgin females as the “WT” reference for these genotypes. Nonvirgin white RNAi ovaries were used for normalizing RNA expression from NSL2 RNAi ovaries. RNA sequencing was conducted using three biological replicates, and the replicates of each genotype showed high concordance (Fig S1D). Total RNA sequencing revealed that NSL1 and NSL3 RNAi resulted in widespread gene misregulation (NSL1 RNAi: 7320 & NSL3 RNAi: 4,053 genes) compared with only 894 genes after NSL2 RNAi (fold change > |2|, *P*-value < 0.05) (Fig 1B, genes in grey). This correlates with the efficiency of the knockdown, that is, *nsl1* and *nsl3* knockdowns are more efficient than those of *nsl2*. All three knockdowns resulted in the up-regulation of transposons (Fig 1C). NSL2 RNAi resulted in the up-regulation of 64 of the 126 transposon families by greater than fivefold. NSL1 and NSL3 RNAi also resulted in the up-regulation of multiple transposon families (Fig 1B). The effects of NSL1, NSL2, and NSL3 knockdown in ovaries on transposon expression showed high overlap and were consistent with the function of these proteins together as a complex (Fig 1E and F). However, NSL1 RNAi and NSL3 RNAi had a stronger overall effect on the transcriptome (grey dots in Fig 1B and G). This global gene misregulation, together with the atrophied morphologies of NSL1 RNAi and NSL3 RNAi ovaries, can result in confounding effects and phenotypes. For the purposes of dissecting NSL complex function in piRNA regulation, we therefore concentrated our subsequent analyses on NSL2 RNAi because these flies carried ovaries which were morphologically normal and could therefore be compared against nonvirgin white RNAi ovaries.

**Figure S2.**
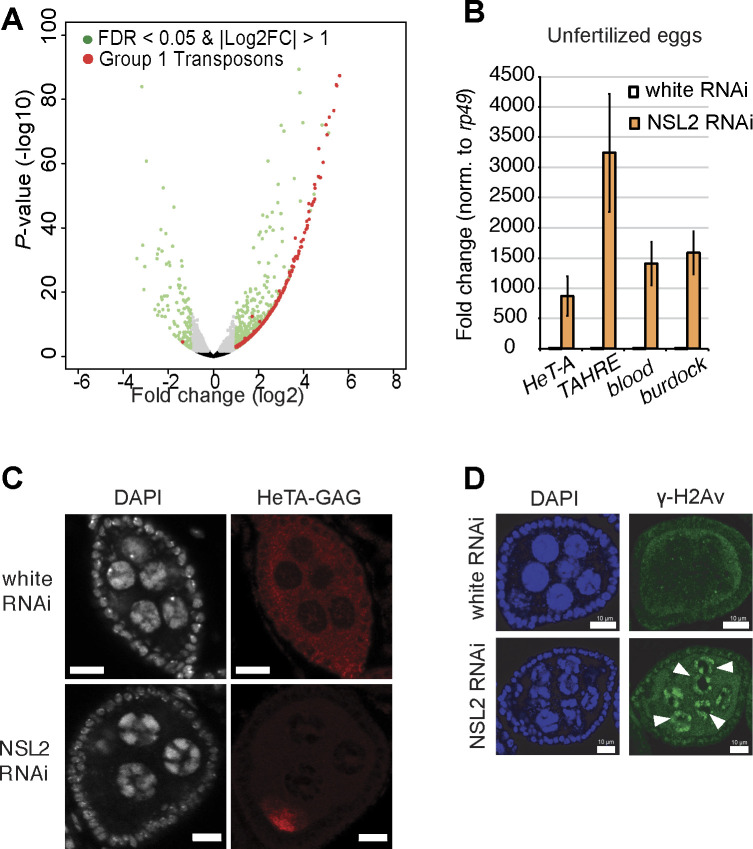
**(A)** Volcano plots showing the log_2_ fold-change and *P*-value of all genes and transposons from the RNA-seq of NSL2 RNAi compared with white RNAi. Data shown are obtained from the DEseq2 analysis of three biological replicates. Shown in green are all genes and transposons with an absolute log_2_FC > 1 and a *P*-value < 0.05. Shown in red are all Group 1 transposons (from Li et al [2009]) with the same requirements. **(B)** Barplot showing RT–qPCR fold changes for transposons *HeT-A*, *TAHRE*, *blood,* and *burdock* in the unfertilized eggs (pure germline RNAs) upon NSL2 RNAi (orange) compared with white RNAi (white). Values were normalized to *rp49*. Values shown are an average of three biological replicates. Error bars represent SD. *piwi P* = 0.49; *vasa P* = 0.33 (paired *t* test). **(C)** Immuno-detection of the HeTA-Gag protein in the ovaries, a translation product of the *HeT-A* transposon, upon white RNAi and NSL2 RNAi. Scale bar, 10 μm. A representative image of n = 6 ovaries is shown. **(D)** Immuno-detection of γ-H2Av protein in white RNAi and NSL2 RNAi ovaries. Arrowheads indicate nurse cells showing accumulation of γ-H2Av. DAPI, 4′,6-diamidino-2-phenylindole. Scale bar, 10 μm. A representative image of n = 6 ovaries is shown.

A previous study defined three broad groups of transposons based on the role played by Ago3 in the production and strandedness (sense:antisense bias) of piRNAs targeting them. Ago3 is required for efficient amplification of piRNAs targeting Group 1 and 2 transposons, but Group 3 transposons are predominantly silenced in an Ago3-independent fashion. Group 1 transposons show a stronger reliance on Ago3 for antisense piRNA amplification, whereas Group 2 shows a more dramatic loss of sense piRNAs upon *ago3* mutation (Li et al, 2009). Group 1 transposons are germline-active transposons, Group 3 are somatic cell-active transposons, and Group 2 are intermediate transposons, that is, active in both germline and somatic cells (Li et al, 2009). We found that predominantly Group 1 (i.e., germline) transposons are up-regulated upon NSL2 RNAi (Figs 1B and D and S2A). *HeT-A* (284-fold) and *HMS-Beagle* (160-fold) were the most up-regulated in the RNA-seq data. None of the Group 2 (i.e., mixed) transposons showed significant up-regulation. Of the Group 3 transposons, only *blood* (283-fold) and *McClintock* (40-fold) transposons with actively transcribed full-length copies in the genome showed up-regulation comparable with Group 1 transposons. We also tested transposon RNA levels in the unfertilized eggs of NSL2 RNAi flies and found >500-fold up-regulation of *HeT-A*, *TAHRE*, *blood*, and *burdock* (Fig S2B), indicating that the up-regulated transposon transcripts are also transmitted to the next generation (Wang et al, 2018).

The transposon derepression observed upon loss of NSL2 also had further consequences for germline cells. It has been shown that the HeTA-GAG protein is expressed and transmitted to the embryos upon piRNA pathway disruption in ovaries (Kordyukova et al, 2018). Immunostaining revealed that the HeTA-GAG protein accumulates in the oocyte of NSL2 RNAi ovarioles (Fig S2C), confirming that up-regulated *HeT-A* transcripts also undergo translation. Furthermore, accumulation of transposons has been shown to cause DNA damage (Klattenhof et al, 2007; Senti et al, 2015; Durdevic et al, 2018; Wang et al, 2018). Thus, we assayed for DNA damage by immunostaining for γ-H2Av, a marker of DNA double-strand breaks. We found that γ-H2Av accumulates in the nurse cell nuclei upon NSL2 RNAi in the ovaries (Fig S2D). Taken together, these data suggest that the NSL complex is crucial for silencing of transposons in the germline of *D. melanogaster*.

### NSL2 is involved in transcription of telomeric piRNA precursors without affecting the ping-pong pathway

**Figure 2.**
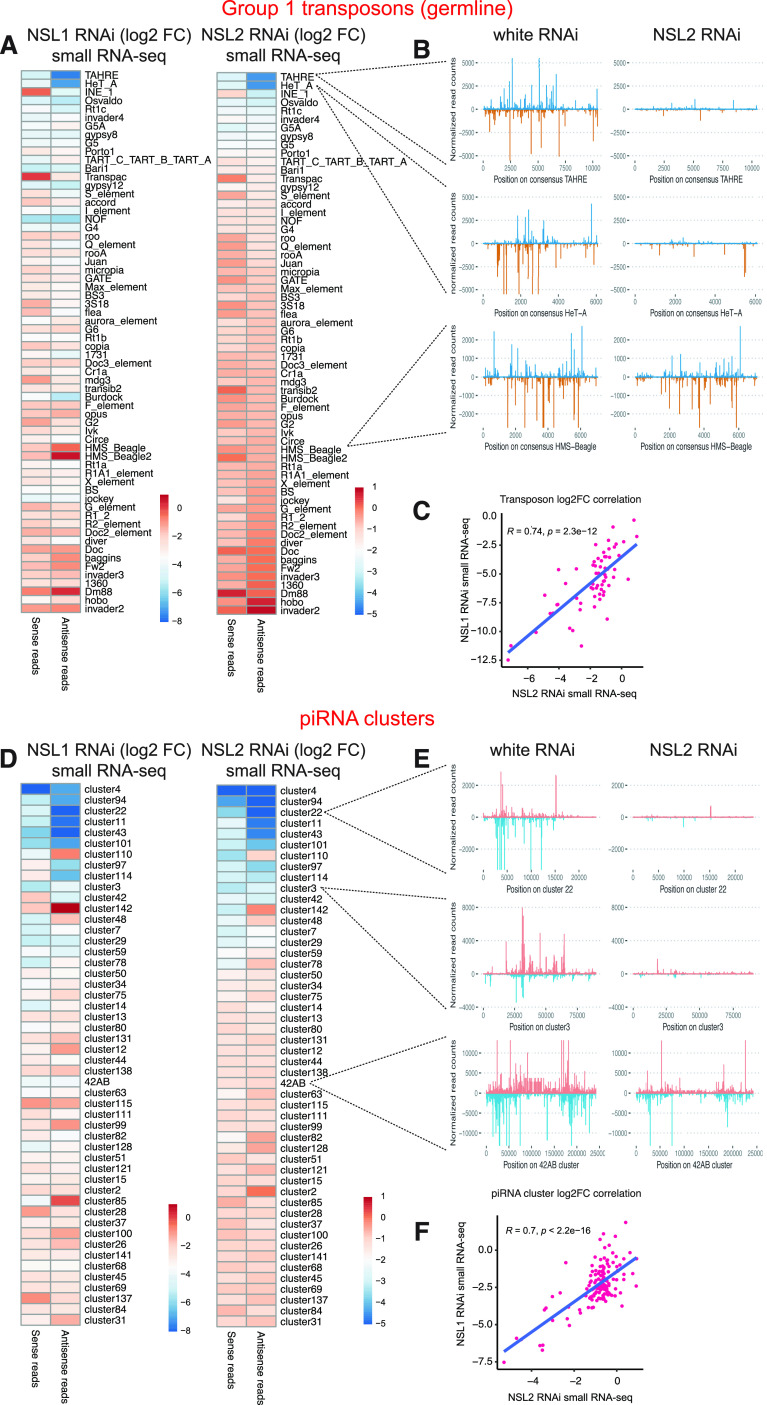
Small RNA sequencing reveals selective loss of piRNAs upon NSL1 or NSL2 depletion. **(A)** Left: heatmap showing log_2_ fold changes of sense and antisense piRNA abundance mapping to Group 1 transposons between NSL1 RNAi and virgin white RNAi ovaries. Right: heatmap showing log_2_ fold changes of sense and antisense piRNA abundance mapping to Group 1 transposons between NSL2 RNAi and white RNAi ovaries. Unaffected transposons are in red or dark orange, down-regulated transposons are in white and pale orange and very strongly down-regulated transposons are in blue. Transposon classification was used from Li et al (2009). Data shown are a representative replicate from two or three replicates with high correlation to each other. **(B)** Sense (blue) and antisense (orange) piRNA abundance over the consensus regions of *TAHRE* (top), *HeT-A* (middle), and *HMS-Beagle* (bottom) is shown for the white RNAi and NSL2 RNAi small RNA-seq data. **(C)** Log_2_ fold changes comparison between NSL1 RNAi and NSL2 RNAi on all Group 1 transposons. *R* represents Pearson correlation. **(D)** Left: heatmap showing log_2_ fold changes of sense and antisense piRNA abundance between NSL1 RNAi and virgin white RNAi mapping to 50 piRNA clusters with the largest changes between NSL2 RNAi and virgin white RNAi. Right: heatmap showing log_2_ fold changes of sense and antisense piRNA abundance between NSL2 RNAi and white RNAi mapping to 50 piRNA clusters with the largest changes between NSL2 RNAi and white RNAi. Data shown are a representative replicate from two or three replicates with high correlation to each other. piRNA clusters showing an increase in mapping piRNAs are in red, showing no change are in dark orange, showing a decrease in white or pale orange, and showing a very strong decrease are in blue. See Table S1. **(E)** Sense (orange) and antisense (blue) piRNA abundance over consensus regions of *cluster 22* (top, telomeric), *cluster 3* (middle, telomeric), and *cluster 42AB* (bottom, pericentric) is shown for the white RNAi and NSL2 RNAi small RNA-seq data. **(F)** Log_2_ fold changes comparison between NSL1 RNAi and NSL2 RNAi on all piRNA clusters. *R* represents Pearson correlation.

**Figure S3.**
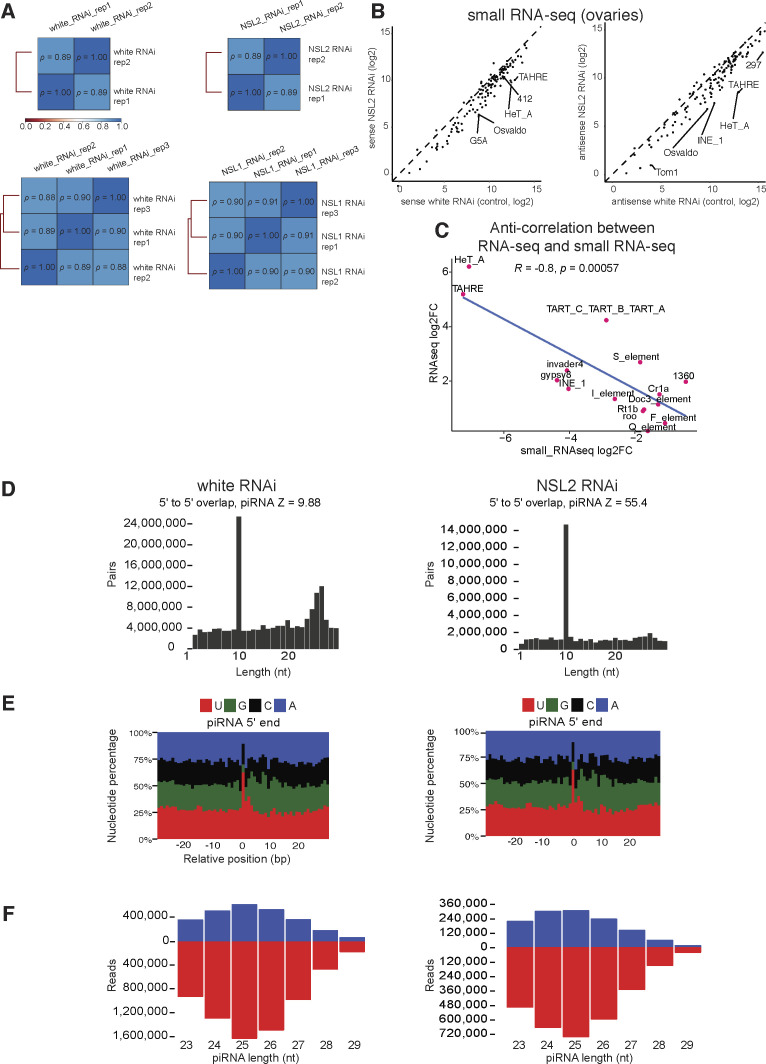
**(A)** Heatmap showing Spearman correlation between small RNA-seq replicates for each sample. **(B)** Scatterplot showing piRNA abundance of sense (left) and antisense (right) reads for white RNAi and NSL2 RNAi ovaries. Selected transposons are labeled. **(C)** Log_2_ fold-change comparison between RNA-seq and small RNA-seq upon depletion of NSL2. *R* represents Pearson correlation. **(D)** Ping-pong analysis for all piRNAs mapping to transposons in white RNAi (left) and NSL2 RNAi ovaries (right). **(E)** Analysis showing the frequency of each nucleotide at each position of every transposon-mapping piRNA in white RNAi (left) and NSL2 RNAi ovaries (right). **(F)** Analysis showing the number of sense (blue) and antisense (red) reads of 23–29 nt length for all transposon-mapping piRNAs in white RNAi (left) and NSL2 RNAi ovaries (right).

Because the piRNA pathway modulates expression of transposons in the germline, we decided to investigate the status of piRNAs by performing small RNA sequencing upon NSL1 RNAi and NSL2 RNAi (Fig 2A). The small RNA sequencing was conducted using biological replicates, with three replicates each of NSL1 RNAi and its corresponding control (virgin white RNAi), and two replicates each of NSL2 RNAi and its corresponding control (white RNAi). The biological replicates of all genotypes showed high concordance (Fig S3A). Initial inspection showed that there was a broad decrease in both sense and antisense piRNA levels (Fig S3B). *HeT-A* and *TAHRE* were the transposons with the strongest depletion of piRNAs (Fig 2A). Mapping the small RNA reads in NSL2 RNAi ovaries onto the consensus sequence of the *HeT-A* and *TAHRE* transposons revealed a very strong depletion of both sense and antisense piRNAs (Fig 2B). Indeed, we also observed a robust global anticorrelation between log_2_FC in piRNAs mapping to a particular transposon family (small RNA-seq) and the log_2_FC of transcripts encoding sequences that map to that transposon family (RNA-seq) when comparing the NSL2 RNAi and white RNAi genotypes (Fig S3C). However, there were some exceptions to this trend. *HMS-Beagle*, a transposon that is up-regulated by >150-fold in the RNA-seq data (Fig 1B), shows little change in piRNAs mapping to it in the small RNA-seq data (Fig 2B). This suggests that the overexpression of *HMS-Beagle* is not because of a decrease in piRNAs, but rather another mechanism. There is a strong correlation between the small RNA reads mapping to transposons in NSL1 RNAi and NSL2 RNAi (Fig 2C).

The transposon classes *HeT-A* and *TAHRE* are prominent in the telomeric clusters, particularly *cluster 3* and *cluster 22*, which contain head-to-tail arrays of HTT transposons, all inserted in an antisense manner. Consistent with the pronounced effect on these telomeric piRNAs, the HTT-containing telomeric piRNA clusters *cluster 3* and *cluster 22* show acute loss of piRNAs mapping to them in both NSL1 RNAi and NSL2 RNAi (Fig 2D and E, log_2_FC −3.3 for *cluster 3* and -4 for *cluster 22* in the NSL2 RNAi). The largest piRNA cluster, *cluster 42AB*, shows a milder loss of piRNAs mapping to it (Fig 2E, log_2_FC −1.1 in the NSL2 RNAi). Another prominent dual-stranded piRNA cluster, *cluster 80F* (also known as cluster 6; this cluster does not appear in Fig 2D as it is not among the 50 most deregulated clusters) showed an even milder piRNA loss (log_2_FC −0.6 in the NSL2 RNAi). There is a strong correlation between the small RNA reads mapping to piRNA clusters in NSL1 RNAi and NSL2 RNAi (Fig 2F). These results suggest that HTT transposons and piRNA clusters at the chromosome ends are particularly sensitive to the loss of NSL1 and NSL2.

Next, we looked at several features of the piRNAs produced in NSL2 RNAi ovaries. We found a strong ping-pong signature even upon NSL2 RNAi, suggesting that the ping-pong pathway functions normally (Fig S3D). piRNAs showed 5′-uridine enrichment in both white RNAi and NSL2 RNAi (Fig S3E). The size distribution of piRNAs is in the 24–29 nt range with a peak around 25 nt in both white RNAi and NSL2 RNAi (Fig S3F). Our data therefore does not find an effect of NSL complex depletion on ping-pong amplification.

**Figure 3.**
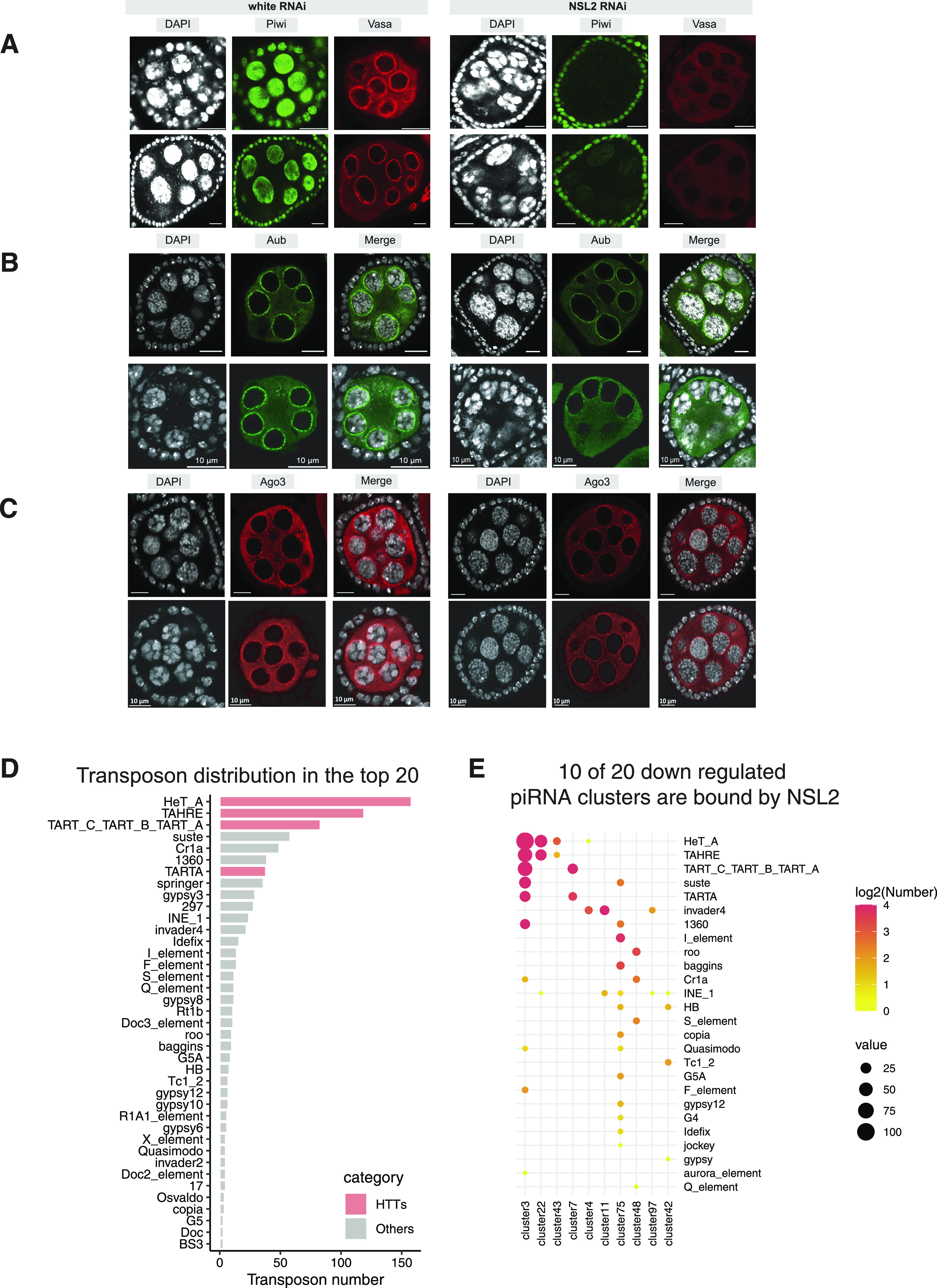
Depletion of NSL2 leads to decreased nuclear Piwi levels. **(A)** Immuno-detection of Piwi and Vasa in white RNAi and NSL2 RNAi ovaries. Scale bar, 10 μm. A representative image of n = 6 ovaries is shown. **(B)** Immuno-detection of Aub in white RNAi (left) and NSL2 RNAi (right) ovaries. Scale bar, 10 μm. A representative image of n = 8 ovaries is shown. **(C)** Immuno-detection of Ago3 in white RNAi (left) and NSL2 RNAi (right) ovaries. Scale bar, 10 μm. A representative image of n = 8 ovaries is shown. **(D)** Barplot showing the total frequency count of full-length or partial transposon insertions belonging to the listed transposon families contained within the 20 piRNA clusters showing the highest changes in piRNA abundance (in other words, most affected) upon NSL2 RNAi (small RNA-seq log_2_FC; see Fig 2D and Table S1). **(D, E)** Dotplot characterizing the transposon composition of piRNA clusters which contain both an NSL2 MACS2 peak and appear in the list of top 20 piRNA clusters showing the most deregulation upon NSL2 RNAi (see panel (D)). The sizes of the bubbles indicate the number of copies of a given transposon element present in that particular piRNA cluster. The colour of the bubble indicates the log_2_ of the number of transposon elements in that particular cluster.

**Figure S4.**
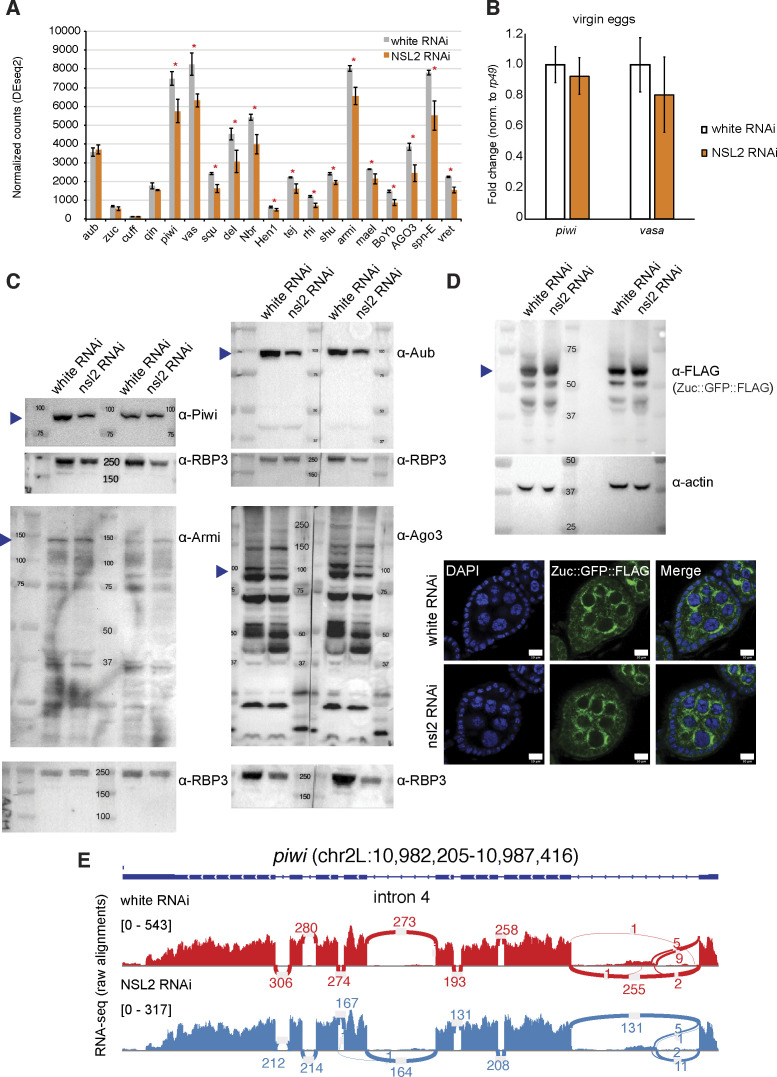
**(A)** Barplot showing the RNA-seq normalized read counts for selected piRNA pathway genes that are important for the regulation of the pathway. Grey bars show values for white RNAi and orange bars show values for NSL2 RNAi. Values shown are an average of three biological replicates. Asterisk denotes a *P*-value < 0.05 (Wald test). Data shown are mean ± S.D. **(B)** Barplot showing RT–qPCR fold changes for *piwi* and *vasa* upon NSL2 RNAi over white RNAi in unfertilized eggs. Values are normalized to housekeeping gene *rp49*. Values shown are an average of three biological replicates. Error bars represent SD. **(C)** Western blots of Piwi, Armi, Aub, and Ago3 from white RNAi and NSL2 RNAi ovaries. Expected sizes of proteins are indicated using arrowheads (Piwi ∼100 kD; Armi 140–150 kD; Aub ∼100 kD; Ago3 ∼100 kD). **(D)** Flies expressing a GFP-FLAG–tagged Zucchini (Pacman BAC clone CH322-41M17 containing the *zuc* locus tagged with GFP-Precission-V5-3xFLAG; Hayashi et al, 2016) were subjected to either white RNAi or NSL2 RNAi. Top: ovary lysates were probed using anti-FLAG. Expected size of the ZUC::GFP::FLAG protein is indicated using an arrowhead (∼56 kD). Bottom: immuno-detection of ZUC::GFP::FLAG in the ovaries. To amplify the GFP signal (to compensate for loss of signal during fixation), the flies were immunostained with anti-GFP^Alexa488^ antibody before imaging. **(E)** Sashimi plot showing the coverage over the *piwi* gene of raw alignments recovered from the RNA-seq from ovaries upon white (red) and NSL2 (blue) RNAi.

Because the piRNA pathway controls transposon silencing in the germline of fly ovaries, we checked the status of Piwi, Vasa, Aub, and Ago3 by immunofluorescence staining. The loss or change in localization of these proteins could provide useful clues about the stage of the piRNA pathway affected by NSL2 depletion. We found that Piwi was severely depleted from the nucleus of the nurse cells upon NSL2 RNAi (Fig 3A). In the same nurse cells, Vasa also failed to localize to the nuage correctly (Fig 3A). The RNA levels of *piwi* and *vasa* RNA in whole ovaries and eggs (Fig S4A and B) and the protein level of Piwi in whole ovaries (Fig S4C) were mildly affected by germline NSL2 depletion. We also noticed that Aub and Ago3 localize correctly to the nuage in NSL2 RNAi ovaries (Fig 3B and C). Their protein levels in the ovary were slightly reduced upon NSL2 RNAi (Fig S4C).

Because the NSL complex is a transcriptional regulator, we checked whether its depletion affects the expression of key genes involved in the piRNA pathway. Several piRNA pathway genes showed reductions at the RNA level, but the changes were mild (Fig S4A). NSL2 depletion also did not appear to affect the perinuclear localization of a GFP-tagged Zucchini construct under the control of its native regulatory region (Hayashi et al, 2016) (Fig S4D). We also observed no change in the protein levels of Armi (Fig S4C). Using the RNA-seq data, we also decided to check the splicing status of *piwi*, because previous reports showed that some transcription regulatory complexes can modify the splicing of intron 4 of *piwi* (Hayashi et al, 2014; Malone et al, 2014). We found no change in *piwi* splicing upon NSL2 RNAi (Fig S4E).

### The NSL complex binds telomeric transposons/piRNA clusters

**Figure S5.**
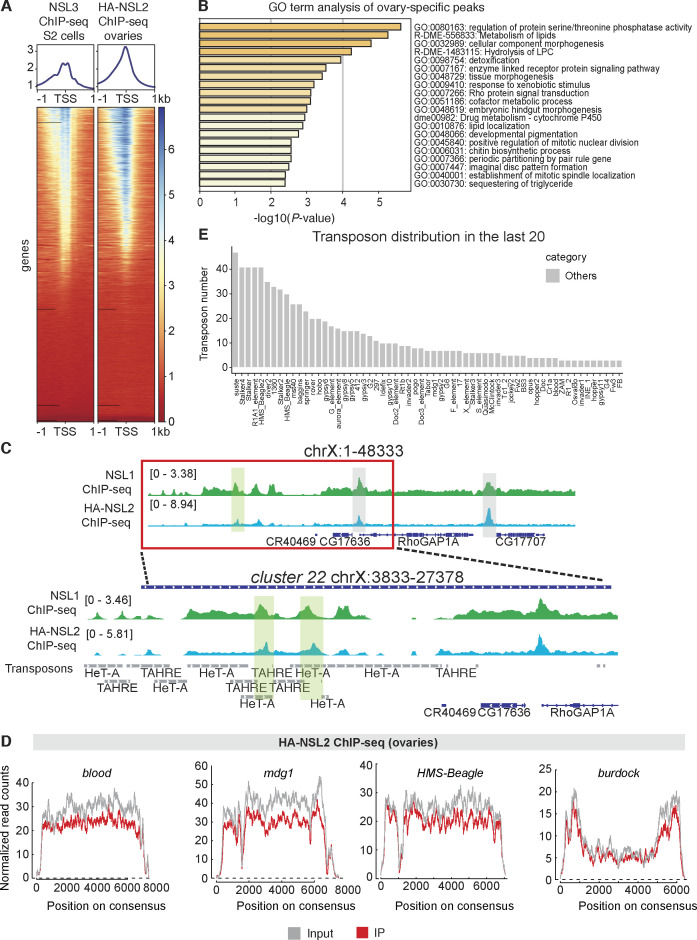
**(A)** Heatmap depicting densities of NSL3 (left, S2 cells) and HA-3xFLAG-NSL2 (right, ovaries) binding over all genes in the fly genome. A profile depicting the average signal is shown above the heatmaps. Data shown are from one representative replicate from two well-correlating biological replicates. NSL3 S2 cell ChIP-sequencing data are derived from Lam et al (2012). **(B)** GO term analysis performed for the genes containing ovary-specific MACS2 peaks from the HA-3xFlag-NSL2 ChIP-seq. **(C)** Genome browser snapshot of the telomeric end of chromosome X showing input-normalized ChIP-seq profiles of NSL1 and HA-3xFLAG-NSL2. The zoomed-in region depicts peaks of NSL1 and NSL2, highlighted in light green shaded boxes, over the telomeric piRNA cluster, *cluster 22*. **(D)** A plot showing density of HA-3xFLAG-NSL2 over the consensus regions of *blood*, *mdg1*, *HMS-Beagle,* and *burdock* obtained by ChIP-seq. The input reads are shown in grey and the immunoprecipitated reads are shown in red. **(E)** Barplot showing the total frequency count of full-length or partial transposon insertions belonging to the listed transposon families contained within the 20 piRNA clusters showing the lowest changes in piRNA abundance (in other words least affected) upon NSL2 RNAi (small RNA-seq log_2_FC; see Fig 2D and Table S1).

Because the NSL complex is a chromatin-associated factor, we wanted to determine the genome-wide binding profiles of NSL complex members in the ovaries. We generated flies carrying endogenous N-terminally epitope-tagged NSL2 (HA-3xFLAG-NSL2) using CRISPR/Cas9. We then separately performed ChIP-sequencing using an antibody against endogenous NSL1 and HA antibody to enrich endogenously tagged HA-3xFLAG-NSL2. To validate that the tagging of NSL2 with HA-3xFLAG does not interfere with its native chromatin localization, we compared the peaks identified in the ovary NSL2 ChIP-seq with previously published data from S2 cells. These datasets showed a large overlap: of the 11,004 peaks identified by MACS2 in ovaries, 10,185 (92.55%) were also identified in S2 cells (Fig S5A). A GO-term analysis of NSL2 targets in the ovary revealed an enrichment for housekeeping functions like lipid metabolism and signal transduction, among others (Fig S5B). We expect these shared genes to represent the tissue-invariant housekeeping targets of the NSL complex.

Table S1. Change in piRNA production from piRNA clusters upon NSL2 RNAi. piRNA clusters are ranked by log_2_FC of sense and antisense (total) piRNAs mapping to that cluster upon NSL2 RNAi in small RNA-seq. The values in columns 1 and 2 represent the raw values underlying the NSL2 RNAi heatmap in Fig 2D.

**Figure 4.**
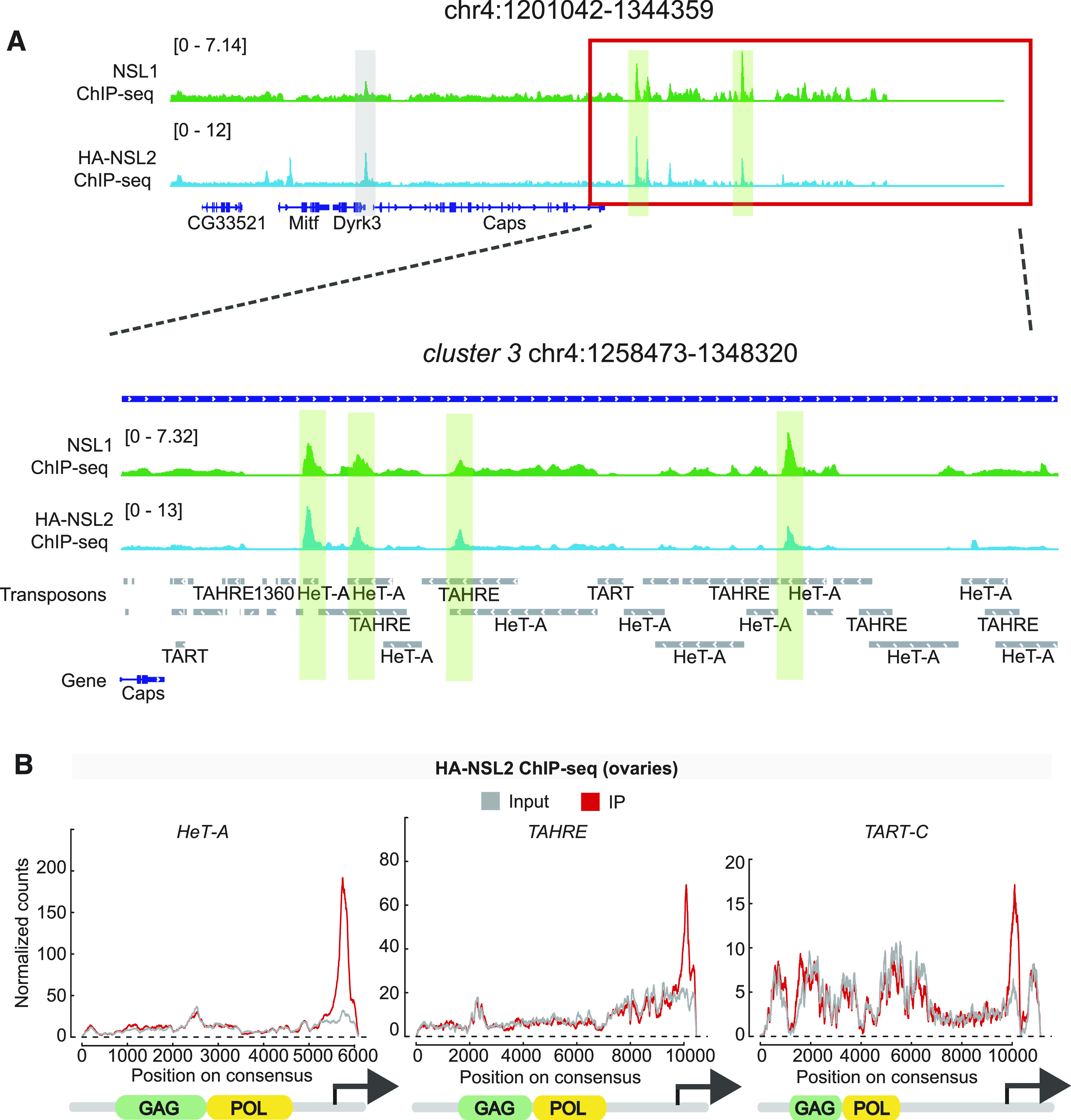
The NSL complex binds to the promoters of telomeric transposons. **(A)** Genome browser snapshot of the telomeric end of chromosome 4 showing input-normalized ChIP-seq profiles of NSL1 and HA-3xFLAG-NSL2. The zoomed-in region depicts peaks of NSL1 and NSL2, highlighted in green, over the telomeric piRNA cluster, *cluster 3*. Data show a merged bigwig of two independent replicates, that is, ovaries collected from females from two separate crosses. **(B)** A plot showing density of HA-3xFLAG-NSL2 over the consensus regions of *HeT-A* (left), *TAHRE* (middle), and *TART-C* (right) obtained by ChIP-seq. The input reads are shown in grey and the immunoprecipitated reads are shown in red. A schematic of the domain structure of each transposon is presented below. The arrow represents the location of the telomeric promoter.

**Figure S6.**
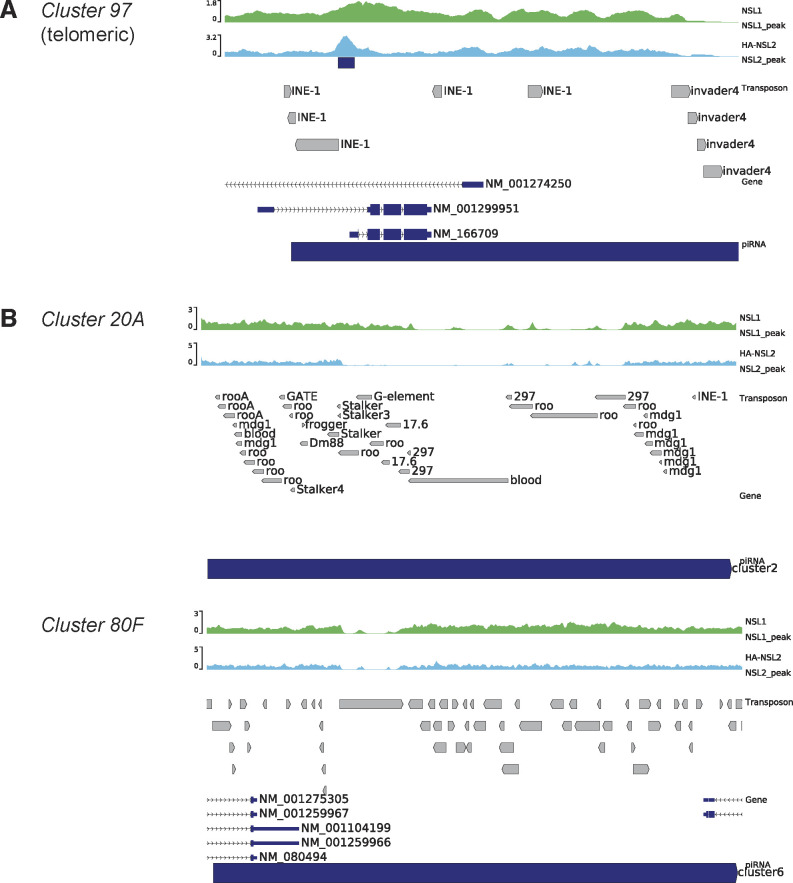
**(A)** Genome browser snapshot of the piRNA *cluster 97* showing input-normalized ChIP-seq profile of HA-3xFLAG-NSL2. This cluster exhibits a MACS2-called NSL2 peak. Data show a merged bigwig of two independent replicates. **(B)** Genome browser snapshot of the piRNA *cluster 20A* and *cluster 80F* showing input-normalized ChIP-seq profile of HA-3xFLAG-NSL2. Note that these two clusters do not exhibit a MACS-identified NSL2 peak. Data show a merged bigwig of two independent replicates.

We next wanted to determine whether piRNA-coding segments of the genome could be bound by the NSL complex. Our small RNA-seq had indicated a significant decrease in piRNAs mapping to telomeric transposons. We therefore went back to our small RNA-seq data to characterize the transposon family composition of the piRNA clusters deregulated by NSL2 depletion. *HeT-A*, *TAHRE,* and *TART* sequences make up the three best-represented (most frequently occurring) transposon families encoded by the 20 most significantly deregulated piRNA clusters in NSL2 RNAi (Fig 3D). On the other hand, no HTT transposons were found in the 20 least deregulated piRNA clusters (Fig S5E). We used our NSL2 ChIP-seq data to identify which piRNA clusters exhibit a MACS2-called peak and compared this against the piRNA clusters showing the highest decrease in piRNA production upon NSL2 RNAi (ranked by log_2_FC of piRNAs mapping to that cluster upon NSL2 RNAi). Half of the piRNA clusters showing the highest decrease in piRNA production (10/20) are bound by NSL2, suggesting that these clusters are likely to be direct targets (Fig 3E). The most deregulated families of transposon in these 10 piRNA clusters were *HeT-A*, *TAHRE*, and *TART*, with half of these putative direct targets (5/10) exhibiting a strong derepression of *HeT-A*, *TAHRE* or *TART*. Genome snapshots showed that both NSL2 and NSL1 bind prominently to *cluster 3* and *cluster 22* (Figs 4A and S5C). Furthermore, telomeric *cluster 97* also showed NSL2 binding (Fig S6B). piRNA production from these three clusters was strongly affected by NSL2 depletion (Fig 2D). On the other hand, *clusters 20A* and *80F* were neither bound by NSL2 nor was their piRNA production strongly affected by NSL2 RNAi (Fig S6B; please note that these clusters are not shown in Fig 2D as they are not among the top 50 most deregulated clusters). Plotting the NSL2 ChIP-seq signal over the consensus sequences of the HTT transposons revealed a pronounced enrichment over the 3′-end of these transposons, that is, over the promoters (Fig 4B). The NSL complex normally binds to promoters located at the 5′-ends of its canonical target genes. HTT transposons, however, contain a promoter at their 3′-terminal regions (Danilevskaya et al, 1997). We checked if the NSL complex binds to other transposons, some of which are known to have a promoter. We found that there is no significant enrichment of the immunoprecipitated reads compared with the input reads over the *blood*, *mdg1*, *HMS-Beagle* or *burdock* consensus sequences (Fig S5D). This result identifies the NSL complex as one of the first protein complexes to exhibit binding to telomeric transposon promoters in *D. melanogaster*.

### Loss of NSL2 alters the chromatin landscape at telomeric piRNA clusters and transposons

**Figure S7.**
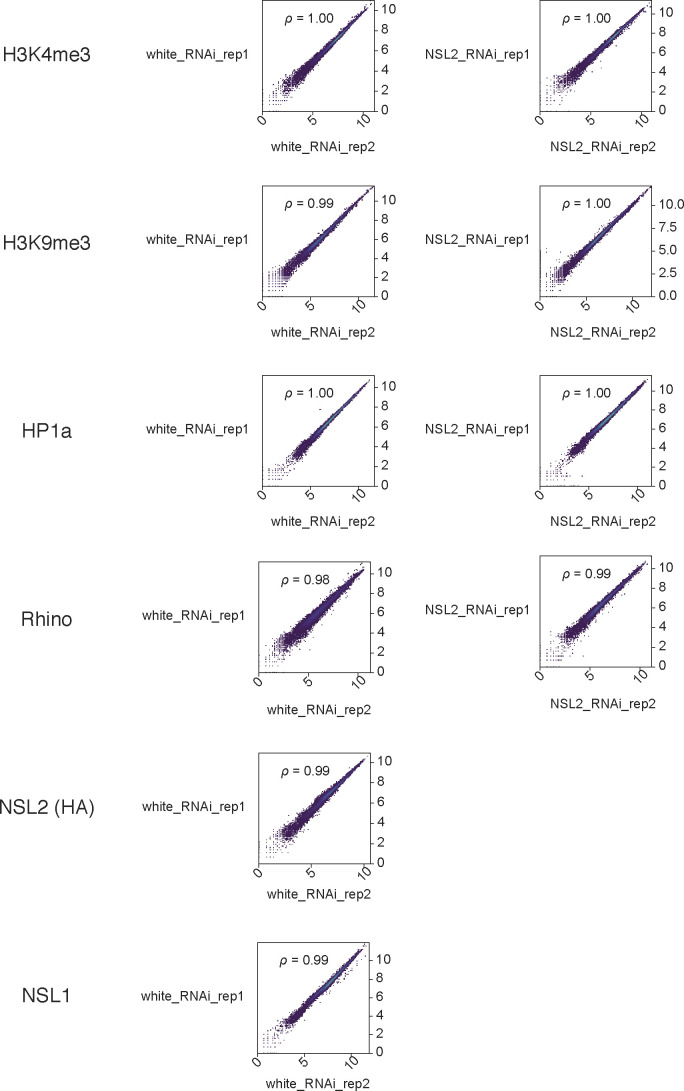
Pairwise scatterplot showing ChIP-seq replicates correlation for each sample.

**Figure S8.**
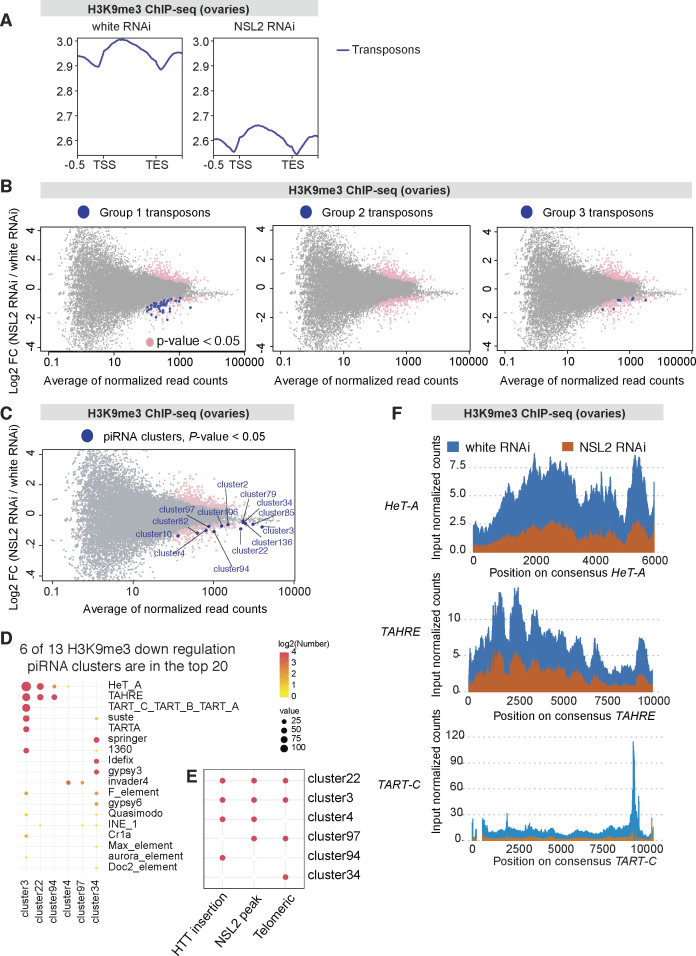
**(A)** Profiles showing average density of H3K9me3 over all transposon insertions in white RNAi and NSL2 RNAi ovaries. Note that the *y*-axis begins at 2.5. TSS- 5′-end of transposon. TES- 3′-end of transposon. **(B)** MA plots depicting the DEseq2 results of ChIP-seq performed to assay the density of H3K9me3 in white RNAi and NSL2 RNAi ovaries. All genes and transposons showing a difference in log_2_ fold-change with a *P*-value < 0.05 are coloured in pink. Transposon insertions showing a difference in log_2_ fold-change with *P*-value < 0.05 are coloured in blue. Group 1 (left), Group 2 (middle), and Group 3 (right) transposons are shown. Data represent an average of three biological replicates. Only unique reads are considered for this analysis. **(C)** MA plots depicting the DEseq2 results of ChIP-seq performed to assay the density of H3K9me3 in white RNAi and NSL2 RNAi ovaries. All genes and transposons showing a difference in log_2_ fold-change with *P*-value < 0.05 are coloured in pink. The 13 piRNA clusters showing a difference in log_2_ fold-change with a *P*-value < 0.05 are coloured in blue. Data represent an average of three biological replicates. Only unique reads are considered for this analysis. **(C, D)** There is an overlap of six piRNA clusters between the 13 piRNA clusters exhibiting statistically significantly reduced H3K9me3 after NSL2 RNAi (from panel (C)) and the top 20 piRNA clusters showing the highest decrease in piRNA production upon NSL2 RNAi (as analyzed in Fig 3D and E). Dotplot showing the number of transposons belonging to the indicated transposon families contained within each of these six piRNA clusters. The sizes of the bubbles indicate the number of copies of a given transposon element present in that particular piRNA cluster. The colour of the bubble indicates the log_2_ of the number of transposon elements in that particular cluster. **(D, E)** Summary of the characteristics of the six piRNA clusters from panel (D). “HTT insertion” indicates the presence of at least 1 *HeT-A*, *TAHRE* or TART transposon insertion within that cluster. NSL2 peak indicates that at least 1 NSL2 ChIP-seq peak is detected within that cluster. “Chromosome tip” indicates that the cluster is located within 70 kb of the chromosome end. **(F)** Density of H3K9me3, obtained from input-normalized ChIP-seq, in white RNAi (blue) and NSL2 RNAi (orange), plotted over consensus regions of telomeric transposons *HeT-A* (top), *TAHRE* (middle), and *TART-C* (bottom).

It is known that the coordination of piRNA cluster transcription, especially the telomeric piRNA clusters, is achieved by the establishment of a unique chromatin state at these sites. Hence, we wanted to dissect the impact of NSL2 loss on the chromatin at telomeric piRNA clusters/transposons. We performed ChIP-sequencing for H3K9me3, which is a hallmark of Piwi-mediated silencing of transposons. All ChIP-sequencing experiments were performed using two biological replicates each, with replicate pairs showing high concordance (Fig S7). Plotting the fold enrichment over the input revealed a global reduction in signal over transposon insertions after NSL2 RNAi (Fig S8A). Upon closer inspection, we realized that most of the decrease in the signal over transposon insertions was on Group 1 transposons (exclusively germline), with 17 of 41 Group 1 transposons showing a significant (*P* < 0.5) decrease in H3K9me3. A decrease of H3K9me3 was observed over a limited number of Group 3 (somatic) transposons but none of the Group 2 transposon insertions (Fig S8B).

**Figure 5.**
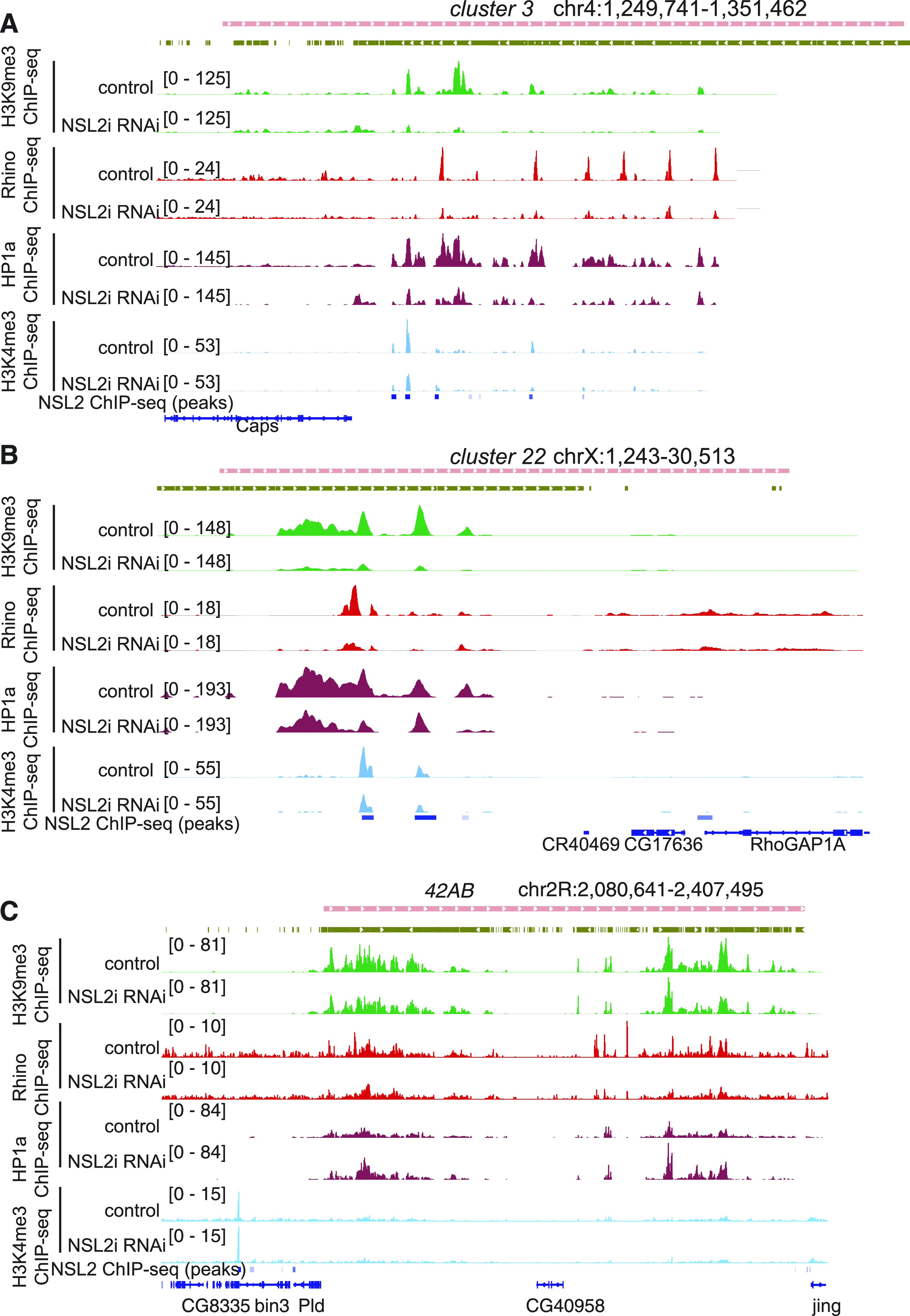
Loss of NSL2 leads to a reduction of H3K9me3, Rhino, HP1a, and H3K4me3 over telomeric piRNA clusters. **(A)** Genome browser snapshot of the piRNA cluster *cluster 3*, showing input-normalized ChIP-seq profiles of H3K9me3 (green), Rhino (red), HP1a (maroon), and H3K4m3 (light blue) upon white and NSL2 RNAi. Blue blocks depict peaks called by MACS2 from the HA-3xFLAG-NSL2 ChIP-seq. Data show a merged bigwig of two independent replicates, that is, ovaries collected from females from two separate crosses. **(B)** Genome browser snapshot of the piRNA cluster *cluster 22*, showing input-normalized ChIP-seq profiles of H3K9me3 (green), Rhino (red), HP1a (maroon), and H3K4m3 (light blue) upon white and NSL2 RNAi. Blue blocks depict peaks called by MACS2 from the HA-3xFLAG-NSL2 ChIP-seq. Data show a merged bigwig of two independent replicates, that is, ovaries collected from females from two separate crosses. **(C)** Genome browser snapshot of the piRNA cluster *42AB*, showing input-normalized ChIP-seq profiles of H3K9me3 (green), Rhino (red), HP1a (maroon), and H3K4m3 (light blue) upon white and NSL2 RNAi. Blue blocks depict peaks called by MACS2 from the HA-3xFLAG-NSL2 ChIP-seq. Data show a merged bigwig of two independent replicates, that is, ovaries collected from females from two separate crosses.

We observed significant decreases in H3K9me3 signal over selected piRNA clusters (Fig S8C). Six of the thirteen piRNA clusters with reduced H3K9me3 carried at least one HTT insertion (Fig S8C and D). Four out of six of these clusters were also bound by NSL2. Lastly, four out of six were located at the chromosome tip (Fig S8E), suggesting that telomeric clusters may be particularly affected. *Cluster 3* and *cluster 22*, found at telomeres of chromosome 4 and chromosome X, respectively, showed a strong decrease in H3K9me3 upon NSL2 RNAi (Fig 5A and B, green tracks). In contrast, the largest piRNA cluster, *42AB*, shows little to no change in H3K9me3 density over it (Fig 5C, green tracks). *Cluster 38C1* also shows no change of H3K9me3. Because telomeric piRNA clusters were affected, we examined the signal over the three telomeric transposons, *HeT-A*, *TAHRE,* and *TART*. All three show a strong reduction in H3K9me3 (Fig S8F).

**Figure S9.**
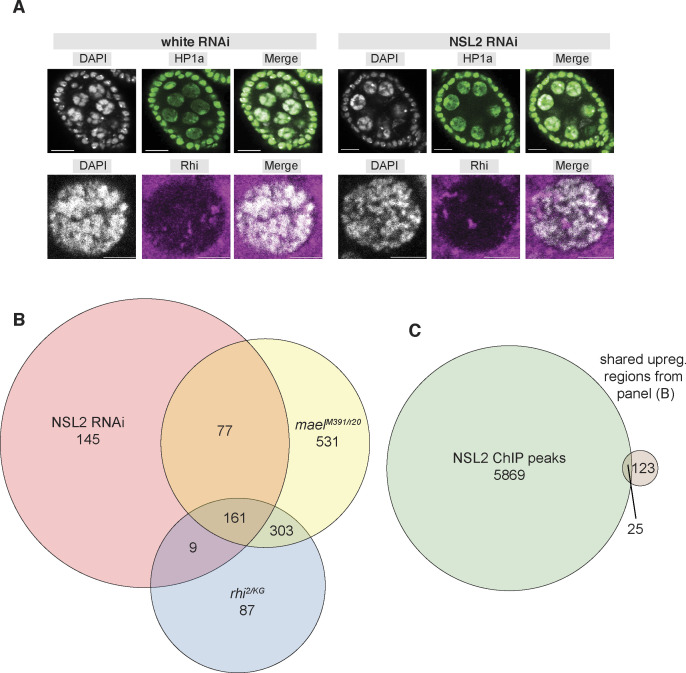
**(A)** Immuno-detection of HP1a upon white RNAi (left) and NSL2 RNAi (right). Scale bar, 10 μm. A representative image of *n* = 5 ovaries is shown per condition. **(B)** Venn diagram showing overlap between transposon elements up-regulated in the NSL2 RNAi, *mael*^*M391/r20*^ and *rhi*^*2/KG*^ mutant ovaries. *mael*^*M391/r20*^ and *rhi*^*2/KG*^ mutant RNA-seq data were taken from Chang et al (2019). **(C)** Venn diagram showing overlap between NSL2 MACS2-called ChIP-seq peaks and transposon elements up-regulated in all three conditions (NSL2 RNAi, *mael*^*M391/r20*^, and *rhi*^*2/KG*^ mutants). **(B)** The common regions are not identical to panel (B) because of the fact that more than one transposon element can overlap with one ChIP-seq peak (in this case it is counted as 1).

To understand the downstream effects of the reduced telomeric H3K9me3 levels elicited by NSL2 depletion, we decided to interrogate the localization of two HP1 paralogs, Rhino and HP1a, upon depletion of the NSL complex. Discrete Rhino foci were observed in oocyte nuclei of both white RNAi and NSL2 RNAi ovaries (Fig S9A). We also found no change in localization of HP1a in NSL2 RNAi ovaries compared with the control (Fig S9A). This was despite a small but significant reduction in the levels of RNA coding for HP1a (*Su(var)205*; log_2_FC −0.308, *P*-value 0.0175) observed upon NSL2 RNAi in our RNA-sequencing data. However, HP1a binding is decreased at both *cluster 22* and *cluster 3*, particularly at sites of NSL2 binding (Fig 5A and B, maroon tracks). This is accompanied by reduced Rhino binding (Fig 5A and B, red tracks). Furthermore, we observed lower H3K4me3 signal at NSL2 peaks on *cluster 22* and *cluster 3* (Fig 5A and B, light blue tracks). We examined the *42AB* cluster and found little to no change of the chromatin landscape (Fig 5C). The NSL complex therefore appears to have compound effects at telomeric piRNA clusters/transposons. The reduced levels of Rhino may result in reduced noncanonical transcription from these sites. Furthermore, the reduction of H3K4me3 hints that loss of NSL2 may also be associated with reduced canonical transcription at the telomeric piRNA clusters/transposons.

**Figure 6.**
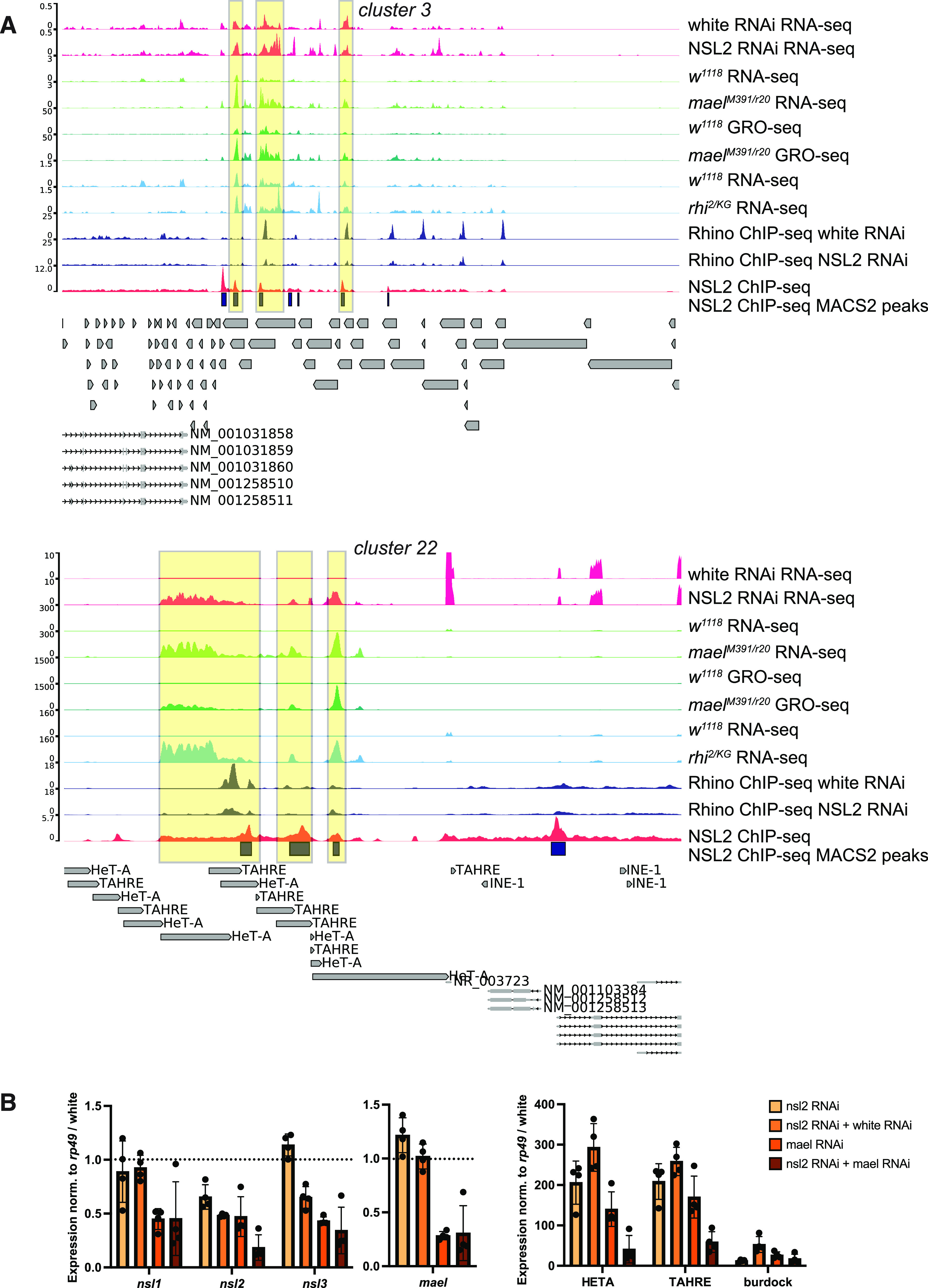
Comparison of NSL2 RNAi with *mael*^*M391/r20*^ and *rhi*^*2/KG*^ RNA-seq data. **(A)** Genome browser snapshots of the piRNA clusters *cluster 3* and *cluster 22*, showing RNA-seq profiles of white RNAi and NSL2 RNAi (magenta); RNA-seq profiles in control *w*^*1118*^ and *mael*^*M391/r20*^ mutant ovaries (light green); GRO-seq profiles of *w*^*1118*^ and *mael*^*M391/r20*^ mutant ovaries (dark green); RNA-seq profiles of *w*^*1118*^ control and *rhi*^*2/KG*^ mutant ovaries (light blue); Rhino ChIP-seq profiles of white RNAi and NSL2 RNAi (dark blue); and NSL2 ChIP-seq profile in WT (red). Dark blue blocks depict peaks called by MACS2 from the HA-3xFLAG-NSL2 ChIP-seq. White RNAi RNA-seq, NSL2 RNAi RNA-seq, NSL2 ChIP-seq, and Rhino ChIP-seq data show a merged bigwig of two independent biological replicates. RNA-seq datasets in *w*^*1118*^ (SRR8078485, SRR8078482, SRR8078483), *mael*^*M391/r20*^ (SRR8078565, SRR8078564, SRR8078563) and *rhi*^*2/KG*^ (SRR8078593, SRR8078594, SRR8078595) mutants and GRO-seq datasets from *w*^*1118*^ (SRR8078585, SRR8078586, SRR8078583) and *mael*^*M391/r20*^ mutants (SRR8078587, SRR8078588, SRR8078581) are from Chang et al (2019). Data show a merged bigwig of three independent replicates. **(B)** RT-qPCR analysis of expression of *mael*, NSL complex members *nsl1*, *nsl2*, *nsl3*, and three classes of transposon (*HeT-A*, *TAHRE*, *Burdock*) in NSL2 RNAi, combined NSL2 + white RNAi, mael RNAi, and combined NSL2 + mael RNAi ovaries. Each bar represents the mean ± SD of four independent biological replicates. All values were normalized first to *rp49* and then to white RNAi (white RNAi level is set at 1 and is indicated by the dotted horizontal lines).

Rhino is involved in promoting both canonical and noncanonical dual-strand piRNA cluster transcription. However, in the WT (Maelstrom-expressing) genetic background, Rhino predominantly mediates noncanonical transcription. This is because canonical piRNA cluster transcription is suppressed by Maelstrom (Chang et al, 2019). Genomic regions whose transcription increases in *mael* null mutants may therefore help reveal sites of canonical transcription. We reanalyzed published RNA-seq datasets in *w*^*1118*^ (WT), *mael*^*M391/r20*^, and *rhi*^*2/KG*^ mutants (Chang et al, 2019) using the same parameters as we used for our own white RNAi and NSL2 RNAi RNA-seq datasets. We also plotted GRO-seq from *w*^*1118*^ and *mael*^*M391/r20*^ mutants (Chang et al, 2019). We observed an overlap in the genomic regions within *clusters 3* and *22* showing up-regulation of transcripts after depletion of NSL2 or loss of *rhino* or *maelstrom* (Fig 6A, yellow-boxed regions). We found a partial overlap between transposon sequences up-regulated upon depletion of NSL2, mutation of *maelstrom* and mutation of *rhino* (Fig S9B). Many of the shared regions were also either at or adjacent to an NSL2 ChIP-seq peak: 16.9% of the 148 up-regulated regions common to NSL2 RNAi, *mael*^*M391/r20*^ and *rhi*^*2/KG*^ mutants are adjacent to a MACS2-called NSL2 peak (Fig S9C).

We set out to test for genetic interaction between *mael* and *nsl2*. Depletion of either *nsl2* or *mael* alone results in significant derepression (>100-fold increase) of both *HeT-A* and *TAHRE*. Combining NSL2 RNAi with *mael* RNAi strongly attenuates this derepression (Fig 6B). It is interesting that *mael* and *nsl2* show a genetic interaction. Published work suggests that *mael*^*M391/r20*^ ovaries show increased H3K4me3 signal at derepressed transposons, including HTTs (Chang et al, 2019). On the other hand, our data indicate that NSL2 depletion results in decreased H3K4me3 at NSL2-bound HTT promoters within telomeric piRNA *clusters 3* and *22* (Fig 5A and B). These data hint toward the idea that although Maelstrom suppresses canonical transcription, NSL2 promotes it. Interestingly, these putative opposing effects ultimately produce the same phenotypes in the RNA-seq data: both *mael*^*M391/r20*^ mutants and NSL2 RNAi ovaries show increased levels of TE transcripts mapping to telomeric piRNA *clusters 3* and *22* (Fig 6A, red and green tracks). This highlights the difficulty in disentangling effects on transcription of piRNA precursors from effects on piRNA-mediated transcriptional silencing at telomeres.

**Figure S10.**
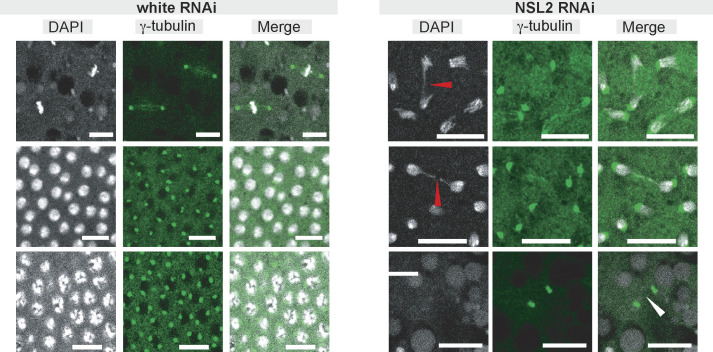
Loss of NSL2 leads to mitotic defects in early embryos. Immuno-detection of γ-tubulin upon white RNAi (left) and NSL2 RNAi (right) in fertilized 0–2-h-old embryos. Scale bar, 10 μm. A representative image of n = 10 eggs is shown.

Because the HTT transposons control telomere maintenance in germline cells of *D. melanogaster*, we decided to investigate whether NSL2 RNAi elicits any defects in telomere maintenance. We used 0−2-h-old embryos laid by control and NSL2 RNAi flies and stained for DAPI and γ-tubulin, a centrosome marker. We already knew that NSL2 RNAi results in lethality of the embryos, with ∼95% of the laid embryos failing to hatch (Fig S1C). One of the hallmarks of telomere dysfunction is the formation of chromosomes or anaphase bridges. Previous studies have demonstrated that *HeT-A* overexpression resulting from germline (*nanos*-GAL4-driven) knockdowns of Ccr4, Not1, Woc, and Trf2 produce mitotic defects in early embryos, including asynchronous division and anaphase bridges, likely caused by telomere fusion (Morgunova et al, 2015). Chromosome bridges, asynchronous mitosis, and sunken nuclei were also observed in HP1a mutant embryos at the nuclear cycle 10–14 stage (Kellum & Alberts, 1995; Fanti et al, 1998; Park et al, 2019). We observed increased incidence of chromosome bridges upon NSL2 RNAi (Fig S10, red arrowheads). Optical sections of the stained embryos also revealed instances of free centrosomes at the periphery, with the nucleus sinking into the interior (Fig S10, white arrowhead). This is highly reminiscent of the phenotype observed in syncytial blastoderm-stage embryos laid by mothers with germline depletion of Ccr4, Not1, Woc, and Trf2, which also exhibited free centrosomes that remained at the cortex, whereas the nuclei sank into the interior of the embryo, leaving regions of the embryo cortex to appear to be devoid of nuclei (Fig S7 of Morgunova et al, 2015). These observations suggest that the NSL complex contributes to the maintenance of telomeres in the oocytes and early embryos through its effects on the piRNA pathway.

## Discussion

### The NSL complex is important for production of telomeric piRNAs

The NSL complex is a conserved regulator of constitutively active genes in somatic cells (Raja et al, 2010; Feller et al, 2012; Lam et al, 2012, 2019). Here, we report that in the female germline, the NSL complex additionally participates in regulation of the piRNA pathway. Nanos-GAL4–driven depletion of *nsl1*, *nsl2* or *nsl3* results in transcriptional derepression of selected transposons (Figs 1B and D) and depletion of *nsl1* or *nsl2* results in decreased levels of piRNAs mapping to multiple transposon families (Fig 2A). At the molecular level, our data suggest that NSL complex depletion likely elicits defects in TE repression through a combination of (1) delocalization/reduced levels of nuclear Piwi and (2) impaired transcription of piRNA precursors from telomeric piRNA clusters. Both of these effects contribute to the phenotypes observed upon NSL complex depletion. NSL2 binding is observed at promoters of *HeT-A*, *TAHRE,* and *TART-C*, and significant reductions in piRNAs targeting them are scored upon NSL1 or NSL2 depletion (Figs 2A and 4B). On the other hand, no NSL2 binding is observed across *blood*, *mdg1*, *HMS-Beagle* or *burdock* elements (Fig S5D). This suggests that the reduction in *blood*-, *burdock*- or *HMS-Beagle*-containing transcripts observed after NSL2 depletion (Fig 1B) is unlikely to be mediated through a transcriptional effect on the loci encoding their piRNA precursors. We also observe the depletion of H3K9me3 over multiple piRNA clusters (Fig S8C), and at telomeric transposons (Fig S8F) after NSL2 depletion. At the transcriptional level, NSL2 RNAi partially phenocopies loss of nuclear Piwi because germline depletion of Piwi produces a dramatic effect on levels of transcripts encoding *HeT-A*, *TAHRE,* and *TART* transposons (Rozhkov et al, 2013; Senti et al, 2015; Yu et al, 2015). However, the chromatin phenotypes of NSL2 knockdown and loss of nuclear Piwi are not identical. Increased H3K4me2 in the first 2 kb of their respective consensus sequences has been reported at multiple transposon families in *piwi*^Nt^/*piwi*^2^ mutants (Fig 3D of Zhang et al, 2021). Mutants which lose nuclear Piwi localization (*piwi*^Nt^/*piwi*^2^) show slightly decreased H3K9me3 but slightly increased H3K4me2 at *HeT-A*, *TAHRE*, and *TART* transposons (Fig S1 of Klenov et al, 2014). NSL2 depletion, on the other hand, results in decreases in both H3K9me3 and H3K4me3 at *cluster 3*, *cluster 22*, and the consensus sequences of *HeT-A* and *TAHRE* transposons (Fig 5A and B and data not shown). Although this interpretation is currently speculative given that we only have H3K4me3 (but not H3K4me2) ChIP-seq data in NSL2 RNAi ovaries, one would anticipate a correlation between these two marks (Ardehali et al, 2011; Mohan et al, 2011). Because *mael* mutants have been reported to partially phenocopy nuclear Piwi loss and Maelstrom is thought to function downstream of Piwi (Sienski et al, 2012; Chang et al, 2019), the genetic interaction between NSL2 and *mael* RNAi (Fig 6B) is another interesting observation, as it cannot easily be explained by loss of Piwi alone. The putative antagonistic relationship between the NSL complex and Maelstrom warrants closer investigation in the future.

### The NSL complex binds the 3′-UTR promoters of HTT transposons

Maintenance of genome integrity is paramount in the germline because it passes on genetic information to the next generation. piRNA-mediated regulation of telomeres serves as an important checkpoint in the development of *D. melanogaster* because telomere-maintaining transposons are extremely sensitive to piRNA loss (Radion et al, 2018) and their overexpression results in the arrest of embryogenesis (Kordyukova & Kalmykova, 2019). Unlike its 5′ TSS binding on its canonical gene targets, at telomeres, NSLs bind to the 3′ promoters of the HTT transposons (Fig 4B). Loss of the NSL complex results changes in the telomeric chromatin, with decreases of both HP1a and Rhino over the telomeric piRNA clusters *3* and *22* (Figs 5A and B). On the other hand, HP1a binding appears to slightly increase after NSL2 RNAi at *cluster 42AB*, although the reason for this is not clear (Fig 5C). In the future, it would be interesting to explore whether there is any interplay between the NSL complex and telomeric maintenance factors such as HOAP.

An outstanding question is how the NSL complex recognizes the promoters of HTT elements. To date, only a couple of factors have been associated with specific regulation of telomeric sequences in Drosophila. *JIL-1*, *Z4*, *Dref*, *Trf2*, and *Ken* mutants were shown to exhibit decreased *HeT-A* and *TART* expression. *Z4* and *pzg* mutants were shown to exhibit telomeric fusions in mitotic chromosomes of third instar larvae (Silva-Sousa et al, 2012). Several unique features distinguish the telomeric piRNA promoters and may make them NSL targets. Whereas other bidirectional piRNA clusters either do not contain or do not rely on canonical promoters for productive piRNA precursor transcription (Mohn et al, 2014), the individual transposons in telomeric clusters each carry canonical promoters and previous work has suggested that the HTT transposons also exhibit higher H3K4me2 enrichment and have a greater tendency to be transcribed than other TEs (Klenov et al, 2014). Our finding that *maelstrom* and *nsl2* show epistasis suggests that these two factors may function in the same pathway as part of the piRNA pathway. Future work will be needed to validate this finding and identify whether NSL2 may promote some level of canonical transcription at selected piRNA clusters by locally counteracting the transcriptional suppression activity of Maelstrom.

### Telomeric transcription in other species

Although HTT TEs only occur in the Drosophila genus, transcription of telomeric and subtelomeric regions is relatively common in eukaryotes (Azzalin et al, 2007; Azzalin & Lingner, 2015). Telomeric transcripts give rise to small RNAs in various species and cell types, including mammalian embryonic stem cells (Savitsky et al, 2006; Cao et al, 2009; Tatsuke et al, 2010; Vrbsky et al, 2010). These studies have demonstrated that telomeric small RNAs contribute to telomeric heterochromatin and telomeric elongation. However, the transcriptional regulation of telomeric transcripts in other species remains poorly understood. In humans, the long noncoding RNA *TERRA* is produced by RNA Pol II-mediated transcription from canonical CG-rich promoters in the subtelomeric regions (Kordyukova & Kalmykova, 2019). This process is regulated by CTCF and the cohesin subunit Rad21 (Azzalin et al, 2007; Deng et al, 2012). Multiple studies indicate that *TERRA* contributes to telomere maintenance (Bettin et al, 2019). More work will be needed to investigate whether the NSL complex also contributes to the transcription of *TERRA* and thereby the maintenance of telomeres in mammals. The cells making up the germline are the most sensitive to mutations, as their genome is transmitted to the progeny. Telomere maintenance and the piRNA pathway work together in the Drosophila ovary to ensure integrity of the genome inherited by the oocyte. Interestingly, through its activity at telomeric transposons/piRNA clusters, the NSL complex appears to contribute to these processes, thereby protecting the genetic material transmitted to the next generation.

## Materials and Methods

### Drosophila husbandry

All flies were reared on a standard cornmeal fly medium at 25°C, 70% relative humidity, and a 12-h dark/12-h light cycle.

The following fly strains were used in the study: Table 1.

**Table 1.** List of fly lines used in this study.

NSL2 RNAi (58162 Bloomington)	*y*^*1*^ *v*^*1*^*; P{TRiP.HMJ22113}attP40*
NSL2 RNAi (46033 VDRC)	*w* ^*1118*^ *; P{GD9504}v46033*
NSL1 RNAi (58328 Bloomington)	*y*^*1*^ *v*^*1*^*; P{TRiP.HMJ22458}attP40/CyO*
NSL1 RNAi (32561 Bloomington)	w[*]; P{w[+mC] = NIG.4699R}3
NSL3 RNAi (v24248 VDRC)	*w* ^*1118*^ *; P{GD13852}v24248*
White RNAi (35573 Bloomington)	*y*^*1*^ sc* v^*1*^*; P{TRiP.GL00094}attP2*
Maelstrom RNAi (34793 Bloomington)	*y*^*1*^ sc* v* sev^*21*^*; P{TRiP.HMS00102}attP2*
*Nanos* (*Nos*)-GAL4 (25751 Bloomington)	*P{UAS-Dcr-2.D}1, w* ^*1118*^ *; P{GAL4-nos.NGT}40*
ZUC::GFP::FLAG (313656 VDRC)	*w; ; Pacman BAC clone CH322-41M17 containing the zuc locus tagged with GFP_Precission_V5_3xFLAG [attP2]/TM3,Sb;*
HA-3xFLAG-NSL2 (generated in this study)	*w; ; endogenous nsl2/dgt1 locus tagged with HA-3xFLAG using CRISPR/Cas9*

Lines 46033 and 32561 were only used in Fig S1.

For assessing the hatching rate of the eggs laid by the female flies upon germline NSL2 RNAi, age-matched females were kept in a cage supplemented with fresh yeast paste with WT males and allowed to lay eggs for 3 h. The eggs were kept at 25°C overnight. Hatched eggs were counted after 30 h. The percentage of hatched eggs of the NSL2 and white RNAi were plotted on a graph.

For assessing the knockdown in the unfertilized eggs, age-matched NSL2 RNAi virgin females were kept in a cage supplemented with fresh yeast paste without any males. They were allowed to lay eggs for 1 h. The eggs were collected and RNA was extracted from these unfertilized eggs.

### RNA isolation

For RNA isolation from ovaries, unfertilized eggs or cell pellets, freshly collected samples were flash frozen in liquid nitrogen and crushed with a nuclease-free pestle (catnum) in a nuclease-free tube. RNA was then extracted using the DirectZol kit (#R2050; Zymo Research) according to the manufacturer’s manual. 5–10 ovaries, up to 200 virgin ovaries, and ∼100 μl of eggs were used per replicate. RNA concentration was determined using a Qubit 2.0 fluorometer (#Q32866; Invitrogen).

### RNA sequencing and analysis

Purified RNA (1 μg) was used to prepare libraries for sequencing. The TrueSeq Stranded Total RNA Library Prep (#20020597; Illumina) was used to generate libraries using the manufacturer’s recommendations. Ribosomal RNAs were depleted using a RiboZero step during the library preparation. The sequencing was done on the HiSeq3000 (Illumina) machine with a sequencing depth of 30–50 million reads per sample.

The analysis was done using piPipes (Han et al, 2015). Briefly, reads were mapped to the Drosophila genome (dm3) using bowtie2 (Langmead & Salzberg, 2012) to rRNA reads first and the unmapped reads were aligned to the genome using STAR RNA-seq aligner (Dobin et al, 2013). Transcripts were counted using featureCounts (Liao et al, 2014) or eXpress (Forster et al, 2013). Differential expression analysis was performed using DESeq2 (Love et al, 2014).

ERCC spike-in normalization was used for the normalization of RNA-seq data from the ovaries (size factors). The ERCC spike-in reads were mapped and quantified in the same way as the other RNAs (STAR→ featureCounts→ DESeq2).

### Quantitative reverse transcription PCR (RT–qPCR)

400 ng of purified RNA was used, for each sample, to prepare the cDNA using the SuperScript III First-Strand Synthesis System (#18080051; Invitrogen) according to the manufacturer’s protocol. Random primers were used for the cDNA preparation. RT-qPCR was performed on this cDNA using primers shown in Table 2. SYBR Green I mastermix (#04707516001; Roche) was used on a Roche LightCycler 480 machine.

**Table 2.** List of primers used in this study.

Name	Sequence
*rp49*_forward	ATGACCATCCGCCCAGCATAC
*rp49*_reverse	CTGCATGAGCAGGACCTCCAG
*TAHRE*_forward	CTGTTGCACAAAGCCAAGAA
*TAHRE*_reverse	GTTGGTAATGTTCGCGTCCT
*HeT-A*_forward	CGCGCGGAACCCATCTTCAGA
*HeT-A*_reverse	CGCCGCAGTCGTTTGGTGAGT
*burdock*_forward	AGGGAAATATTTGGCCATCC
*burdock*_reverse	TTTTGGCCCTGTAAACCTTG
*blood*_forward	CCAACAAAGAGGCAAGACcG
*blood*_reverse	TCGAGCTGCTTACGCATACTGTC
*piwi*_forward	TTACCCGTACTTCGTCCTGATG
*piwi*_reverse	TTGGGCACCGAAATAACTCA
*nsl1*_forward	AGGAAAACCCTACCCGATGT
*nsl1*_reverse	ATTCCCATCTAGCTGGCTGA
*nsl2*_forward	TAGCTGATCGTGAATGCTGC
*nsl2*_reverse	ATTGCCGAAACGAAGCTGAT
*nsl3*_forward	AAAACCATCTCCTGCATGGG
*nsl3*_reverse	ACGAGGAGCTGTCAGAGATT
*vasa*_forward	TGTAGTGATGTTCTGGACGC
*vasa*_reverse	AATGTCTGATGTTCTGGACGC
*mael*_forward	CTC GTG CTA AAC GCC AAG AT
*mael*_reverse	ATA GAC GTC GGT GGT CAA GG
3′-adapter (AppBA3, 21-nt custom DNA oligo with the 5′adenylation and the 3′ddC)	5′-rAppTGGAATTCTCGGGTGCCAAGG/ddC/-3′
5′-adapter (BA5, 26-nt customRNA oligo, PAGE purified)	5′-GUUCAGAGUUCUACAGUCCGACGAUC-3′

Two or more technical replicates were used for each sample. The fold changes were derived using the 2^(ΔΔCt) method (Livak & Schmittgen, 2001).

Primers used for RT-qPCR are listed in Table 2.

### Chromatin immunoprecipitation followed by deep sequencing (ChIP-seq)

For ChIP from ovaries, 200 freshly dissected ovaries were collected in a tube. For ChIP from OSS cells, 20–50 million cells per replicate were pelleted. The samples were then crushed in 100 μl of Buffer A1 (60 mM KCl, 15 mM NaCl, 4 mM MgCl_2_, 15 mM HEPES pH 7.6, 0.5% TritonX-100, 0.5 mM DTT, protease-inhibitor cocktail) + 1% formaldehyde for crosslinking with a plastic pestle. The solution was transferred into a 1 ml glass dounce homogenizer and 900 μl of Buffer A1 + 1% formaldehyde was added. The mixture was dounced with the tight pestle for 20 strokes. It was then transferred to a rotating wheel at room temperature. After 20 min from the start of crosslinking, the solution was quenched by adding 160 μl of 2.5 M glycine for 5 min. The mixture was then centrifuged at 4,000*g* for 5 min and the supernatant was discarded. The pellet was resuspended in 1 ml of Buffer A1 and centrifuged again for 4,000*g* for 5 min. This step was repeated twice. After the last round of centrifugation, the pellet was resuspended in 1 ml of lysis buffer (140 mM NaCl, 15 mM HEPES pH 7.6, 1 mM EDTA pH 8, 0.5 mM EGTA, 1% TritonX-100, 0.5 mM DTT, 0.1% sodium deoxycholate, protease inhibitor cocktail) supplemented with 0.5% SDS and 0.5% N-laurylsarcosine. This was kept in a rotating wheel at 4°C for 1 h.

The mixture was split into 5 x 200 μL and sonicated using Covaris E220 (peak power 150, duty cycle 10, cycles/burst 200). Chromatin of fragment size 300–600 bp was obtained. After centrifugation at 10,000*g* for 5 min, the supernatant containing the sheared chromatin was transferred into a new tube. The chromatin was precleared with 50 μl 50:50 slurry of Sepharose Prot A/G beads (Invitrogen) overnight at 4°C.

10% of the sheared chromatin was used as the input. To the remaining 90% antibody (indicated in Table 3) was added and incubated at 4°C overnight. The next day, 50 μl 50:50 slurry of Sepharose Prot A/G beads was added and incubated at 4°C for 3 h to immunoprecipitate the antibody. The beads were collected by centrifugation and the supernatant was either discarded or stored for checking the quality of shearing/sonication. The beads were washed 4x with lysis buffer supplemented with 0.05% SDS for 5 min each time at 4°C. After this, the beads were washed 2x with TE buffer (10 mM Tris–HCl pH 8, 0.1 mM EDTA) for 5 min each time. Then, 1 ml of TE buffer was added and the samples were decrosslinked overnight at 65°C, 1,400 rpm in an Eppendorf ThermoMixer. The following day, 1 μl of RNase A (10 mg/ml) was added and the samples were incubated at 37°C for 30 min. Then, 5 μl of 10% SDS and 1 μl of Proteinase K (10 mg/ml) were added and the samples incubated at 50°C for 90 min. DNA was extracted using the ChIP DNA Clean and Concentrator Kit (D5205; Zymo Research). The DNA was quantified using Qubit 2.0 fluorometer. Libraries were prepared using the NEBNext Ultra2 library preparation kit (#E7645; New England Biolabs) according to the manufacturer’s protocol. The libraries were sequenced on the HiSeq3000 machine and 75 bp long reads were obtained. About 10–20 million paired-end reads per sample were obtained.

**Table 3.** List of antibodies used for immunofluorescence staining (IF) and Western blotting (WB).

anti-HA	Mouse	1:400 (IF)	901501 (BioLegend)
anti-FLAG^HRP^	mouse	1:10,000 (WB)	clone M2, A8592 (Sigma-Aldrich)
anti-Piwi	mouse	1:2000 (IF), 1:1,000 (WB)	Gift from Dr. PD Zamore
anti-GFP^Alexa488^	rabbit	1:500 (IF)	Invitrogen (A-21311)
anti-γ-H2Av	mouse	1:300 (IF)	UNC93-5.2.1 (DSHB)
anti-Vasa	rat	1:300 (IF)	AB_760351 (DSHB)
anti-Aub	mouse	1:2,500 (IF), 1:1,000 (WB)	Gift from Dr. PD Zamore
anti-Ago3	rabbit	1:500 (IF), 1:500 (WB)	Gift from Dr. PD Zamore
anti-Rhino	guinea pig	1:300 (IF)	Gift from Prof. WE Theurkauf
anti-HP1a	mouse	1:300 (IF)	C1A9 (DSHB)
anti-gamma-Tub	mouse	1:200 (IF)	Sigma-Aldrich (T6557)
anti-Armi	mouse	1:1,000 (WB)	clone 2F8A9, gift from Prof. M Siomi
anti-actin^HRP^	mouse	1:2,000 (WB)	Santa Cruz (sc-47778 HRP)
anti-RBP3	rabbit	1:1,000 (WB)	own lab stock
a-HeTA-GAG	guinea pig	1:500 (IF)	Gift from Prof. Y Rong

### ChIP-seq analysis

The analysis was done using piPipes (Han et al, 2015). Briefly, the paired-end reads were mapped to the Drosophila genome (dm3) using bowtie2 using the -u option for reporting unique mappers only. The resulting BAM files of the replicates were merged for all the downstream analysis. Peaks were called the MACS2 tool (Feng et al, 2012). Bigwig files were generated using deepTools2 (Ramírez et al, 2016) using the log_2_ fold change of ChIP/Input or using MACS2 (poisson value option). Enrichment of signal over TSS of dm3 genes were calculated by checking for the signal from the bigwig files over ±200 bp from the TSS using the computeMatrix function of deeptools2. Heatmaps were made using the output from the computeMatrix output. IGV genome browser (Thorvaldsdóttir et al, 2013) was used to visualize the signal over many regions of the genome.

### Small RNA-sequencing

15 μg of purified total RNA was used for each replicate of small RNA sequencing. For isolating small RNAs ranging from 18–29 nt, a 12.5% 6–8 M urea gel was cast. Total RNA diluted to 1 μg/μl and an equal volume of formamide loading buffer (#R0641; Thermo Fisher Scientific) was added. The sample was heat denatured at 95°C before being loaded into the gels. An 18-nt and a 30-nt RNA marker mixture was also loaded to aid in isolating the 18–29 nt RNAs. The gels were run at 15–20 W for 45 min. The gels were stained with 1x SYBR Gold for 5 min and the gel was excised above the 18-nt marker and below the 30-nt marker. The gel piece was placed in a tube and the RNA was eluted overnight with 1.2 ml of 0.3 M NaCl.

The following day, the supernatant was split into three parts and the RNA was precipitated using 500 μl of 100% ethanol on ice for 1 h. The sample was then centrifuged at 17,000*g* for 15 min and the supernatant was discarded. 900 μl of 75% ethanol was added and the sample was vortexed briefly before centrifugation at 17,000*g* for 5 min. The supernatant was discarded and the pellet was air-dried for 2 min. The pellet was resuspended in 8 μl of nuclease-free water.

After this, the 3′-adapter (see Table 2) was ligated to the small RNAs and the ligation reaction was allowed to run for 16 h at 25°C. Then, the RNA was treated with phenol/chloroform, the upper layer was treated with 3 M sodium acetate pH 5.2, and the RNA was precipitated using 100% ethanol on ice for 1 h. The RNA pellet was resuspended in 13 μl water and 1 μl of 10 μM 2S block oligo (to remove any remaining 2S rRNA).

After this, the 5′-adapter (Table 2) was ligated to the sample using T4 RNA ligase (Ambion). The RNA was isolated as before and resuspended in 13 μl of water. The resulting RNA was reverse-transcribed using AMV Reverse Transcriptase (NEB) for cDNA. The cDNA was amplified using AccuPrime Pfx DNA Polymerase (#12344024; Invitrogen) for 13 cycles. The libraries were then gel purified from a 2% agarose gel using the QIAgen Gel Extraction Kit. The DNA concentration was checked on the DNA bioanalyser and the samples were sequenced on the HiSeq3000 platform to obtain 30–40 million reads per sample.

### Small RNA-sequencing analysis

The analysis was done using piPipes (Han et al, 2015). Briefly, the reads were mapped to rRNAs first and the unmapped reads were mapped to miRNA hairpin database, siRNA database, repbase-annotated transposons, and piRNA clusters using bowtie. BEDtools (Quinlan & Hall, 2010) were used to assign the reads to different annotations and eXpress was used to quantify them. This quantification was used to plot the piRNA abundance over consensus transposons and over piRNA clusters.

Multiple normalization strategies were used to normalize the samples. The data presented here are normalized to the number of unique non-rRNA reads in each library.

Then the small RNAs were separated into different sizes based on lengths. The number of unique piRNA pairs with their 5′-ends exactly 10 nt away from each other were calculated and plotted.

### Immunostaining of embryos

Briefly, embryos were aged at 25°C until they reached the desired developmental stage. After dechorionation using 50% bleach, embryos were fixed in a mix containing equal volumes of heptane and 4% formaldehyde in PBS for 20 min under vigorous shaking at RT. This step was skipped when aiming for histone modification immunostaining. The formaldehyde solution was removed and methanol was added to the heptane solution in equal volume. Intense shaking for 40 s leads to the dissociation of the vitelline membrane and further fixation. The embryos that sank after shaking were collected and rinsed three times with methanol to remove any traces of formaldehyde. The embryos were then rehydrated using PBS-0.1% Triton solution for 15 min three consecutive times. Blocking was achieved by incubation with PBS-Triton 0.1% supplemented with 0.2% BSA for 30 min. Antibodies used are mentioned in Table 3. Images were captured on the LSM780 confocal microscope (Carl Zeiss Microscopy) using an alpha Plan-Apochromat 63x/1.4 (DIC) oil objective.

### Immunofluorescence in ovaries

8–10 fly ovaries were freshly dissected in 1X PBS. The excess supernatant was removed and 800 μl of fixing solution was added (4% formaldehyde in PBS). The tube was left at room temperature for 17 min with occasional flipping.

After fixing, the ovaries were rinsed with 1X PBS three times. Then they were washed with 1X PBS for 15 min at room temperature. Then they were rinsed three times with a washing solution (0.2% TritonX-100 in PBS). Then they were washed 2X for 15 min each time with the washing solution at room temperature.

The ovaries were then blocked with a blocking solution (0.2% TritonX-100, 1% bovine serum albumin in PBS) for 30 min at room temperature.

The samples were then incubated with primary antibodies of choice (Table 3) in the blocking solution overnight at 4°C in a rotating wheel. The next day, they were rinsed 3X with the washing solution and then washed with the washing solution 3X for 15 min each time. Then the species-appropriate secondary antibody (in blocking solution) was added and the samples were incubated for 1 h at room temperature with mild shaking. Then the samples were rinsed 3X with the washing solution. Then the samples were washed 3X with the washing solution for 15 min each time. The individual ovarioles were mounted on Vectashield or ProLong Gold (#P36930; Invitrogen) and the slides were ready for imaging.

Images were captured on the LSM780 or AiryScan confocal microscopes (Carl Zeiss Microscopy) using an alpha Plan-Apochromat 63x/1.4 (DIC) oil objective.

### Western blotting on whole ovaries

10 fly ovaries were freshly dissected in 1X PBS. The excess supernatant was removed and 100 μl of 2X SDS sample buffer (K929.1; Carl Roth) was added. The ovaries were homogenized using a micropestle and boiled at 95°C for 2 min 10 μl of the sample was run on a NuPAGE 4–12% Bis-Tris PAGE gel (NP0322PK2; Thermo Fisher Scientific) using 1X MOPS running buffer. After separation, proteins were transferred to a 0.45 μm PVDF membrane using a wet transfer chamber held under constant voltage set to 100 V for 90 min at 4°C. The membrane was blocked for 1 h in dilution buffer (5% milk powder in 1X PBS, 0.2% Tween-20). Primary antibodies were diluted in dilution buffer at the indicated concentration (Table 3). Non-HRP-conjugated primary antibodies incubated at overnight at 4°C. HRP-conjugated primary antibodies were incubated for 1 h at room temperature. Membranes were washed 3 × 5 min in washing buffer (1X PBS, 0.5% Tween-20). For HRP-conjugated primary antibodies, blots were developed at this stage. For non-HRP-conjugated primary antibodies, membranes were incubated with species-appropriate HRP-conjugated secondary antibodies in dilution buffer at room temperature for 1 h. Subsequently, the membranes were again washed 3 × 5 min in washing buffer and then developed using the Lumi-Light (12015200001; Roche) chemiluminescence substrate.

### Data visualization

The scatterplot, heatmap, and PCA plot of replicates were produced with deepTools multiBamSummary, multiBigwigSummary, plotCorrelation, and plotPCA (v3.5.0) (Ramírez et al, 2016). The Venn diagrams were plotted with the eulerr package (https://cran.r-project.org/web/packages/eulerr/index.html). The scatterplot for log_2_ fold change correlation, barplot, and dotplot were produced with ggplot2 (v3.3.2; https://ggplot2.tidyverse.org) and heatmaps of expression changes were produced with pheatmap (v1.0.12) in R (v 4.0.3). The representative tracks were produced by pyGenomeTracks (v3.5.1) (Lopez-Delisle et al, 2021) and IGV (Robinson et al, 2011).

## Data Availability

The RNA-sequencing and ChIP-sequencing datasets generated during this study have been deposited in the Gene Expression Omnibus (GEO) database as accession GSE156897. RNA-seq data in *w*^*1118*^, *mael*^*M391/r20*^, and *rhi*^*2/KG*^ ovaries; and GRO-seq data in *w*^*1118*^ and *mael*^*M391/r20*^ ovaries from Chang et al (2019) are available from the Sequence Read Archive (SRA) via accessions SRR8078485, SRR8078482, SRR8078483, SRR8078565, SRR8078564, SRR8078563, SRR8078593, SRR8078594, SRR8078595, SRR8078585, SRR8078586, SRR8078583, SRR8078587, SRR8078588, SRR8078581. RNA-sequencing data from S2 cells subjected to NSL1 or NSL3 RNAi from Gaub et al (2020) are available at GEO via accession GSE135815. ChIP-sequencing data for NSL3 in S2 cells have been published previously (Lam et al, 2012) and are accessible in the ArrayExpress database via accession E-MTAB-1085.

## Supplementary Material

Reviewer comments
